# Comparative Transcriptomics Reveals an Extracellular Worm Argonaute as an Ancestral Regulator of LTR Retrotransposons

**DOI:** 10.1093/gbe/evag117

**Published:** 2026-05-08

**Authors:** Isaac Martinez-Ugalde, Kyriaki Neophytou, Yenetzi Villagrana-Pacheco, Adriana Orrego Durañona, Lewis Stevens, Xiaochen Du, Rowan Bancroft, Jessica L Hall, Amy B Pedersen, Mark Blaxter, Amy H Buck, Cei Abreu-Goodger

**Affiliations:** Institute of Ecology and Evolution, School of Biological Sciences, University of Edinburgh, Edinburgh, UK; Department of Molecular Genetics, Faculty of Medicine, University of Toronto, Toronto, ON, Canada; Institute of Immunology & Infection Research, School of Biological Sciences, University of Edinburgh, Edinburgh, UK; Institute of Ecology and Evolution, School of Biological Sciences, University of Edinburgh, Edinburgh, UK; Institute of Cell Biology, School of Biological Sciences, The University of Edinburgh, Edinburgh, UK; Tree of Life, Wellcome Sanger Institute, Hinxton, UK; Institute of Immunology & Infection Research, School of Biological Sciences, University of Edinburgh, Edinburgh, UK; Institute of Ecology and Evolution, School of Biological Sciences, University of Edinburgh, Edinburgh, UK; Institute of Ecology and Evolution, School of Biological Sciences, University of Edinburgh, Edinburgh, UK; Institute of Ecology and Evolution, School of Biological Sciences, University of Edinburgh, Edinburgh, UK; Tree of Life, Wellcome Sanger Institute, Hinxton, UK; Institute of Immunology & Infection Research, School of Biological Sciences, University of Edinburgh, Edinburgh, UK; Institute of Ecology and Evolution, School of Biological Sciences, University of Edinburgh, Edinburgh, UK

**Keywords:** argonaute, parasite, retrotransposons, RNA interference, RNA communication

## Abstract

Safeguarding the genome from non-self-elements is essential for development, reproduction, and aging. One of the major threats to genomic integrity is transposable elements (TEs), which can be post-transcriptionally silenced through small RNAs (sRNAs) and argonaute proteins. Recent work suggests TE-derived sRNAs may also act as virulence factors in host–pathogen interactions. During infection, the intestinal parasite *Heligmosomoides bakeri* secretes a single argonaute protein (exWAGO) and a wide variety of TE-derived sRNAs. Although exWAGO is highly expressed, conserved, and secreted by parasitic nematodes, its function and sRNA guide preference remain unclear. Using comparative transcriptomics of the sRNAs bound to exWAGO within parasites of rodents, livestock, and humans, and its orthologs in *C. elegans*, we found that exWAGO is capable of loading sRNAs produced from all classes of TEs in addition to some protein-coding and noncoding transcripts. However, our results suggest that the ancestral endogenous function of exWAGO was likely linked to LTR retrotransposon regulation. To understand how this relates to potential extracellular functions of exWAGO, we also examined the sRNAs bound to exWAGO secreted by *H. bakeri* in both vesicular and nonvesicular forms. Extracellular exWAGO preferentially loads sRNA guides derived from nonautonomous and fragmented LTRs, suggesting the existence of adaptable reservoirs of regulatory sRNAs with potential roles in cross-species RNA communication. Together, our results show that exWAGO is part of an evolutionarily conserved pathway for LTR retrotransposon regulation, while preferentially utilizing degenerated elements as sources of secreted sRNAs.

Significance Parasitic worms manipulate the immune responses of their hosts by secreting a variety of molecules during infection, including small RNAs. It has been unclear whether these RNAs are connected to processes that also operate within the worms and how this system evolved. We show that LTR retrotransposons are a conserved source of secreted small RNAs, revealing a link between an ancient genome-defense pathway and parasite-host interactions.

## Introduction

Although typically neutral (Arkhipova 2018), transposable elements (TE) can have deleterious effects on their host genomes, such as gene disruption or ectopic recombination. Disregulated TE activity can also induce genome expansion, potentially leading to genomic instability. To offset this, host genomes have adapted different molecular mechanisms, including noncoding RNA pathways, to regulate TE activity (Wolf et al. 2020; Zhou et al. 2020; Van Lopik et al. 2023). The PIWI-interacting RNA (piRNA) pathway, for instance, is conserved across metazoans (Calcino et al. 2018) and plays a key role in TE silencing through sequence-specific targeting. In *Caenorhabditis elegans,* the PIWI argonaute (PRG-1) and its bound piRNAs trigger TE silencing by inducing the amplification of antisense secondary small interfering RNAs (known as 22G siRNAs) by RNA-dependent RNA polymerases, which are loaded by worm-specific argonautes (WAGOs) (reviewed by Fischer 2024). These WAGO-22G complexes are ultimately responsible for TE silencing. Many clades of nematodes, however, have independently lost essential components of the PIWI pathway, including either the PIWI argonaute protein or the capacity to produce piRNAs (Sarkies et al. 2015; Beltran et al. 2019). Clade V nematodes (divided into Strongylida and Rhabditina) have mostly maintained PIWI argonaute and the capacity to produce piRNAs. Strongylida parasites, however, show oversized genomes with large TE content, compared to the free-living Rhabditina (International Helminth Genomes Consortium 2019). Despite the presence of the PIWI pathway in these nematodes, little is known about how TEs are regulated beyond *C. elegans*.

The gastrointestinal parasite of mice *Heligmosomoides bakeri* secretes immunosuppressive extracellular vesicles (EVs) during infection, which carry TE-derived 22G siRNAs and the extracellular worm argonaute (exWAGO) (Buck et al. 2014; Chow et al. 2019). *H. bakeri* is closely related to human infective nematodes (including the human hookworms *Necator americanus* and *Ancylostoma* spp.), which collectively affect close to 25% of the human population (World Health Organization 2020; Stevens et al. 2023). Given the limited anthelmintic options and the increase in resistance to them, novel therapeutic options are required. We recently showed that *H. bakeri* secretes exWAGO, both inside EVs and in a vesicle-free form, and that immunization against exWAGO confers partial protection against *H. bakeri* infection (Neophytou et al. 2026). Both exWAGO forms associate with TE-derived small RNAs and can be internalized by host cells, suggesting a potential role in RNA-based cross-species communication (Neophytou et al. 2026). Furthermore, exWAGO is conserved among clade V nematodes and highly expressed relative to other argonaute proteins (Chow et al. 2019). Biochemical and proteomic analyses show its orthologs can be secreted by different species, including *C. elegans*, human-infective *Ancylostoma ceylanicum*, ruminant-infective *Teladorsagia circumcincta* and *Trichostrongylus colubriformis*, and rat-infective *Nippostrongylus brasiliensis* (Chow et al. 2019; Nikonorova et al. 2022; Rooney et al. 2022; Uzoechi et al. 2023; Neophytou et al. 2026). While the role of exWAGO in the extracellular environment is potentially linked to extracellular communication, including parasite–host communication, its endogenous function and the features that drive its preference for loading specific sRNA guides remain uncharacterized.

Here, we use comparative genomics and transcriptomics of exWAGO-bound sRNAs in clade V nematodes to investigate how exWAGO guide preference evolved, and to probe how this underlies both genome defense and extracellular RNA export. Our findings suggest that the ancestral role of exWAGO was to regulate LTR retrotransposons. We also identify specific properties of secreted LTR-derived sRNAs in *H. bakeri,* highlighting how nonautonomous LTR fragments are a preferential source for potential cross-species RNA communication functions in parasitic nematodes.

## Results

### Improving Transposable Element Annotation in Strongylida Genomes

We previously observed that exWAGO prefers transposon-associated siRNAs (Howe et al. 2017; Chow et al. 2019; Neophytou et al. 2026). Yet to properly understand transposon regulation, we need high-quality annotations of genomic repeats. To gain an evolutionary perspective on transposon regulation, we first refined the repeat annotations for the *H. bakeri*, *H. polygyrus*, *N. brasiliensis*, *T. circumcincta* and *A. ceylanicum* genomes, using de novo prediction followed by automatic and manual curation (see Methods: Transposable element annotation).

**Fig. 1.**
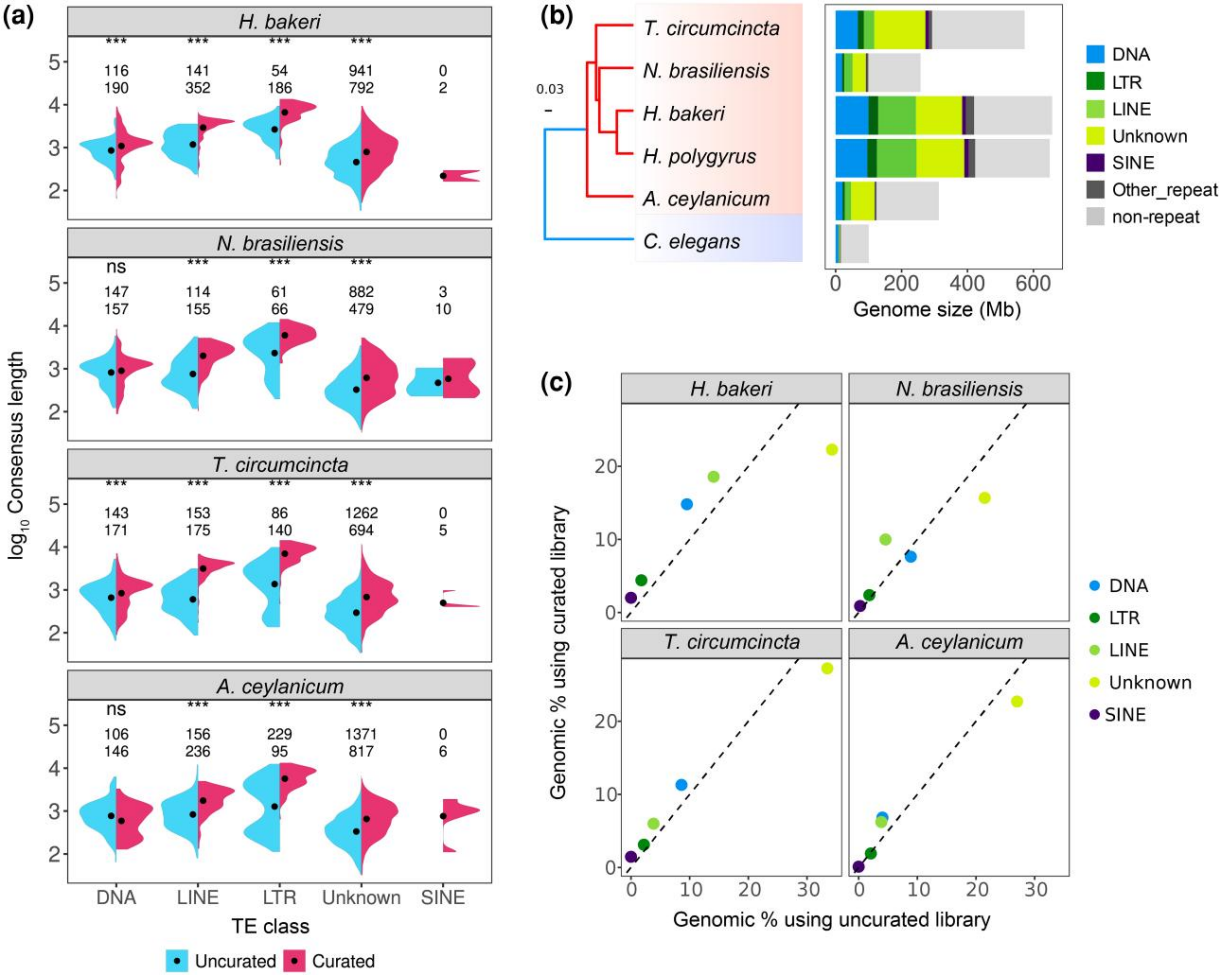
Improved repeat annotations for Strongylida genomes. a) Comparison of the number and length of uncurated and curated consensus models, by TE category. A one-tailed Wilcoxon Rank Sum test was used for comparisons (ns not-significant; * *P*-value < 0.05). The first/second row of numbers indicates the number of non-redundant uncurated/curated models. b) Phylogenetic relationship and repeat content in the analyzed species based on a maximum likelihood phylogeny inferred with 443 BUSCO genes. The repeat span is shown in megabases (Mb). c) Comparison of the percentage of annotated bases per TE category between curated and uncurated libraries. The dotted line denotes x = y.

To assess if our curated repeat libraries improved the quality of genome annotations, we compared them against available repeat libraries from WormBase ParaSite and previous annotation projects (Howe et al. 2017; Chow et al. 2019). Given the lack of a previous repeat library, *H. polygyrus* was excluded from this comparison. We first compared the number and length of nonredundant consensus sequences of the most common TE categories: DNA transposons (DNA), long interspersed nuclear elements (LINEs), long terminal repeat retrotransposons (LTRs), short interspersed nuclear elements (SINEs), and Unknown repeats (Unknown). Our curated libraries in general contain a similar or increased number of consensus sequences for DNA, LINE, and LTR elements, but a decrease in Unknown repeats, suggesting the latter have been incorporated into named classes (Fig. 1a). Our curated models also show a significant increase in length regardless of the category (one-tailed Wilcoxon rank-sum test, *P* < 0.01) (Fig. 1a). Finally, regardless of TE classification, curated models encode a larger number of relevant protein domains (eg transposase, reverse transcriptase) (Table S1). Our curated models are thus not only longer but will also enhance functional analyses of TEs in these organisms.

We further assessed the degree of similarity between uncurated and curated libraries using the benchmarking method provided by RepeatModeler2 (Flynn et al. 2020). Briefly, this benchmark uses a tested library to annotate a reference library, to then classify models based on their relative sequence similarity, coverage, and divergence into perfect, good, present, and missed models.

For *H. bakeri*, when comparing the uncurated against the curated library, we found 60 families with perfect agreement, 278 good, 509 present, and 876 missing. This means that 876/1,726 (51%) of the models in the curated library are missing in the uncurated library. We found similar proportions of missing models in the uncurated libraries for the rest of the species (mean = 39.8%, SD = 7.6%) (Table S2). The number of missing families could reflect highly fragmented/divergent or genuinely absent families in the uncurated sets relative to the curated ones. Together, these results highlight important differences in the coverage and identity level between curated and uncurated libraries.

Our newly annotated genomes vary substantially in repeat content, consistent with their size differences, from 38% of the 257 Mb genome of *N. brasiliensis* to 65% of the 649 Mb genome of *H. bakeri* (Fig. 1b). Notably, the TE span in Strongylida is up to 4 times greater than the entire genome of *C. elegans* (100 Mb), underscoring substantial TE expansion in parasitic lineages.

Importantly, using our curated libraries, we substantially reduce the percentage of bases annotated as Unknown repeats in all the analyzed species by 17% to 22% (Table S3). For instance, in *H. bakeri,* Unknown repeats cover 34.2% of the genome (222 Mb) when using the uncurated library, while only 22.2% (144 Mb) when using the curated library (Fig. 1c). The reduction in bases annotated as Unknown is accompanied by an increase in all the other categories. For example, DNA transposons increase from 9.5% (61 Mb) to 14.7% (96 Mb), LINEs from 14% (91 Mb) to 18.5% (120 Mb) and LTRs from 1.7% (11 Mb) to 4.4% (28 Mb). Although we were able to decrease the span of Unknown repeats in the 4 species we compared (Table S3), it is important to note that Unknown are still the most abundant category of repeats (Fig. 1c), highlighting the value of further curation and characterization efforts.

The improvement in the classification of repeats is particularly meaningful since we previously highlighted Unknown repeats as relevant contributors of exWAGO guides in *H. bakeri* (referred to as Novel repeats in Chow et al. 2019). By refining repeat annotations, we not only increase the accuracy of TE classification but also provide the foundation to improve our understanding of exWAGO guides.

### Improving the Detection of exWAGO-Bound sRNAs

To interrogate the sRNA guides bound to exWAGO in different Strongylida parasites, we used sRNA sequencing libraries generated following immunoprecipitation of exWAGO from adult worms. ExWAGO was pulled down using rat polyclonal anti-exWAGO antibodies raised against recombinant *H. bakeri* exWAGO (for *H. bakeri*, *H. polygyrus*, *N. brasiliensis,* and *A. ceylanicum*) or recombinant *T. circumcincta* exWAGO (for *T. circumcincta*) (Neophytou et al. 2026). Western blot analyses show that immunoprecipitations (IP) were successful, but pull-down efficiency varied for the different species (Neophytou et al. 2026). We generated sRNA libraries from the eluate of the IP and total worm lysates using RNA 5′ polyphosphatase treatment to allow capture of 5′ triphosphorylated sRNAs (Neophytou et al. 2026). These sRNAs show a consistent length (22 to 23 nt length) and 5′ first nt bias (guanine) (Fig. S1). To be able to compare against the binding preference of a free-living rhabditid species, we used published IP data for the exWAGO orthologs in *C. elegans*: SAGO-1, SAGO-2, and PPW-1 (Seroussi et al. 2023).

**Fig. 2.**
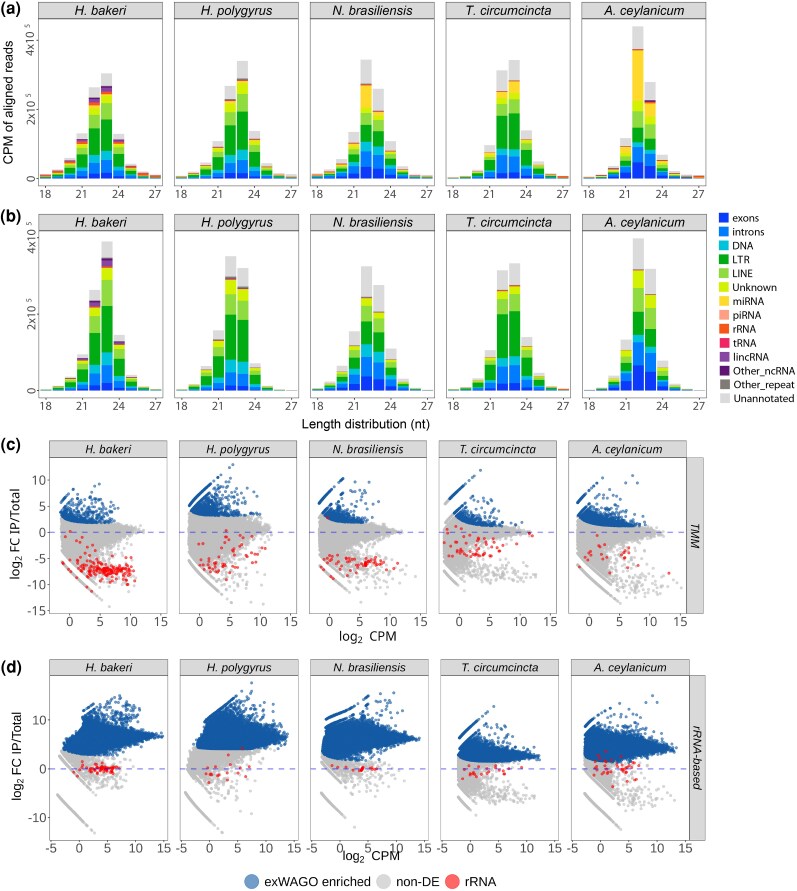
Analysis of exWAGO guides. a) Length distribution of aligned reads by annotation category for Adult total libraries in Strongylida parasites. b) As in (a) but for exWAGO IP libraries. The *x*-axis shows the length of aligned sRNAs (18 to 27 nt), while the *y*-axis shows average counts per million (CPM) across replicate libraries. c) MA plots (logCPM vs. logFC) after differential expression analysis of exWAGO IP against total libraries using TMM normalization. d) As in (c) but using housekeeping (rRNA loci) normalization. Each dot represents a non-overlapping region in each genome. Genomic regions identified as significantly higher in exWAGO IP versus Adult total (FDR < 0.01, see Methods) and interpreted as being bound to exWAGO are highlighted as exWAGO enriched. Non-differentially expressed regions (non-DE) and rRNAs are also indicated. Dashed lines indicate logFC = zero in each contrast.

Sequenced reads from IP experiments represent a subset of the total sRNA population in adult worms (adult total), showing similar length distribution and genomic origin (Fig. 2a/b). To determine which sRNAs are enriched in exWAGO, we quantified the sRNAs mapping to each annotated genomic region for exWAGO-IP and adult worm lysate libraries. We then performed a differential expression analysis, comparing IP to adult total (see Methods). Regions with significantly more sRNAs in the IP, which we call IP-enriched, should represent regions that produce siRNAs specifically bound by exWAGO, while regions with similar amounts of sRNAs in the IP and the input include unbound RNA fragments and those that are non-specifically bound. Initially, we used edgeR with the default Trimmed Mean of M-values normalization (TMM). With this approach, we found 612, 1,591, 978, 1,006, and 3,488 IP-enriched regions for *H. bakeri*, *H. polygyrus*, *N. brasiliensis*, *T. circumcincta*, and *A. ceylanicum*, respectively. For *C. elegans,* we found 4,216, 13,363, and 1,413 IP-enriched regions for SAGO-1, SAGO-2, and PPW-1, respectively. However, the log_2_ Fold-Change (logFC) values show a bimodal distribution, the higher mode likely containing exWAGO-bound sRNA and the lower mode likely representing unbound sRNA (Fig. 2c and Fig. S2a). The default normalization (TMM) is based on the hypothesis that most regions do not change expression, and in this case centers the higher mode (the likely exWAGO-bound sRNAs) on logFC = zero, since it contains more regions than the lower mode. This suggests that “most regions” are actually producing exWAGO-bound sRNAs, with the unbound regions representing a relative minority. Hence, the default normalization method (TMM) cancels this biological effect, thereby hindering the detection of true argonaute-associated sRNAs.

To address this problem, we turned to a “housekeeping” normalization approach (Bullard et al. 2010). This relies on estimating normalization factors using a set of housekeeping genes (those expected not to change in expression) so that the logFC values of this subset of genes are centered on zero. In our case, we had to choose sRNAs that are expected not to be bound by argonaute proteins. Thus, we used the sRNAs mapping to the sense-strand of rRNA genes to estimate normalization factors (see Methods: Differential expression and enrichment analyses of exWAGO and adult total across clade V nematodes). We assume that these rRNA fragments are likely degradation products, and we expect most of them not to be specifically bound by argonaute proteins (Wynant et al. 2017; Zagoskin et al. 2022; Seroussi et al. 2023). To allow for the possibility that some rRNA fragments might be specifically bound and to reduce the effect of outliers, we applied the TMM principle to the rRNA counts to estimate more stable normalization factors.

After applying the new normalization, rRNA genes are consistently centered around logFC = 0 (Fig. 2d and Fig. S2b), and we detect 148,185, 96,605, 140,238, 189,676, and 133,022 exWAGO-bound regions as being significantly enriched for *H. bakeri*, *H. polygyrus*, *N. brasiliensis*, *T. circumcincta,* and *A. ceylanicum*, respectively. Notably, these represent 3.2% to 9.3% of the total number of genomic regions in these genomes (Table S4). These results suggest that in Strongylida, exWAGO binds sRNAs from a large number of genomic regions. Importantly, the regions that we determine not to be bound by exWAGO show a shift toward miRNA and other ncRNA after housekeeping normalization (Fig. S3). Nonetheless, it is important to notice that even after using rRNA to estimate normalization factors, some of the rRNA genes do fall within the exWAGO-bound regions, which could represent real rRNA-derived guides or reflect technical issues due to higher background and/or differing IP efficiency among samples.

Interestingly, when applying the same normalization to the *C. elegans* orthologs of exWAGO, we do not see as substantial a change in enriched regions for SAGO-1 (4,216 to 3,943) or SAGO-2 (13,363 to 14,482), but for PPW-1 the number of enriched regions increases from 1,413 to 42,236 (Fig. S2b and Table S5), similar to the increase for exWAGO. We further analyzed other *C. elegans* argonaute (AGO) IP data (Seroussi et al. 2023), and we observed a large increase in the number of enriched regions for a few WAGOs, including WAGO-1 and PPW-2 (58,357 and 38,286, respectively), but not for other AGOs such as PRG-1 or ERGO-1 (Figs. S4 and S5, and Table S5). These results align with the expected behavior of TMM, which centers the expression values where the majority of regions are distributed (Bullard et al. 2010). Therefore, in AGOs that mostly bind to specific types of sRNA, like ALG-1 or PRG-1, which bind miRNAs or piRNAs, respectively, most of the regions will be unbound. In contrast, AGOs that bind sRNAs from a broader number of genomic regions, as often occurs for siRNAs, we expect an increase in enriched regions when applying rRNA-based normalization.

Finally, to check that our new analyses were not biased toward an increase in regions while sacrificing the quality of these, we quantified the sRNA abundance of other ncRNA species. We found that most regions annotated as ncRNA family species, such as tRNA, miRNA, snRNA, or snoRNA, remain unbound by exWAGO (Table S6). On average, 69.8% of tRNAs remain unbound (SD = 24.4%), 86.1% of miRNAs (SD = 12.2%), 88.1% of snRNA (SD = 2.6%), and 93.5% of snoRNAs (SD = 7.9%). These data indicate that our rRNA-based normalization results maintain biologically meaningful patterns, since WAGOs in general are not expected to bind these ncRNAs (Seroussi et al. 2023).

Interestingly, piRNAs are barely detectable in our adult-worm libraries even though they are reliably annotated for *H. bakeri* and *N. brasiliensis* (Beltran et al. 2019). Moreover, neither exWAGO orthologs in parasites nor *C. elegans* orthologs showed piRNA enrichment, the majority of annotated loci appeared in the unbound fraction (mean = 94%) (Table S6).

Taken together, our results indicate that exWAGO can bind sRNAs from a wide variety of genomic regions. This is consistent with what is observed for other WAGO proteins in *C. elegans* (Seroussi et al. 2023).

### ExWAGO as a Global Regulator of Transposable Elements

**Fig. 3.**
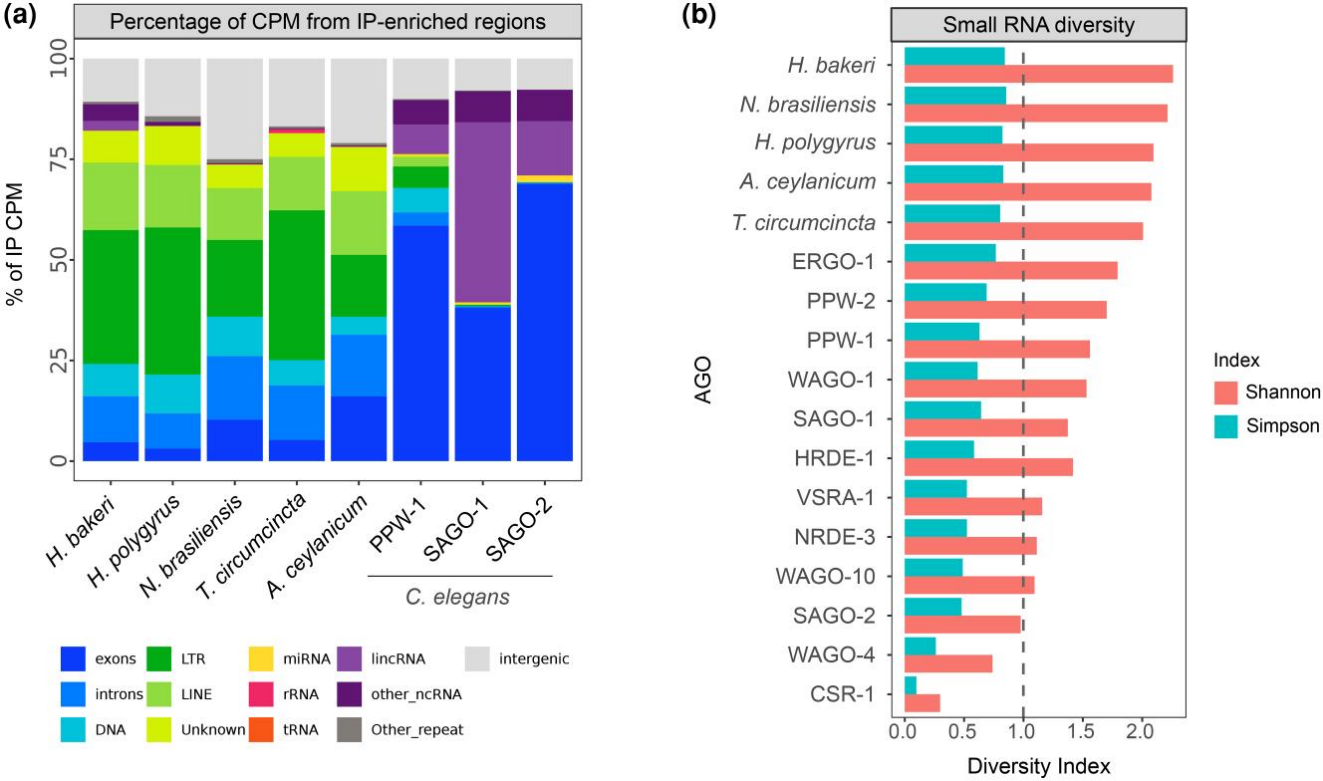
Small RNA diversity in *C. elegans* WAGOs and exWAGO in Strongylida parasites. a) A comparison of the percentage of Counts Per Million (CPM) of enriched regions per genomic category after differential expression analysis. b) Small RNA diversity represented by Shannon and Simpson indices in *C. elegans* WAGOs and exWAGO in Strongylida parasites. The grey dotted line indicates the maximum value (1) of the Simpson index.

Since exWAGO-bound sRNAs map to a large number of regions in each genome, we next asked if exWAGO shows specificity for certain types of annotation. For both *Heligmosomoides* species and *T. circumcincta*, 62% to 71% of the exWAGO-bound sRNA counts are derived from repetitive elements, while for *A. ceylanicum* and *N. brasiliensis,* this value is just under 48% (Fig. 3a). In *C. elegans,* this number is further reduced, where out of the 3 orthologs, only PPW-1 shows more than 14% IP-enriched sRNAs derived from repeats. The opposite trend is observed for protein-coding regions, where the *C. elegans* orthologs show 38% to 68% of sRNA counts, while in *Strongylida,* only 3% to 12% of their exWAGO-bound sRNAs are derived from protein-coding regions. We further categorized sRNAs based on whether they mapped to the sense or antisense strand of each annotation. Antisense strand-derived sRNAs that map to exons, DNA transposons, LTR retrotransposons, LINEs and lincRNAs, are consistently more abundant than those derived from the sense strand (Fig. S6). This is consistent with WAGO guides being produced by RNA-dependent RNA polymerases (RdRps) using a primary RNA as a template (Pak and Fire 2007; Sarkies et al. 2015).

Notably, in Strongylida parasites, 98% to 100% of the TE families with detectable sRNAs in the Adult libraries have at least one copy enriched in exWAGO IP. This includes DNA transposons, LTRs, LINEs, SINEs, and Unknown repeats, suggesting that exWAGO could regulate most of the expressed TEs in these parasites. Similarly, the *C. elegans* ortholog PPW-1 binds sRNAs from 99% of the TE families with detectable sRNA production. Even though SAGO-1 and SAGO-2 have a shared evolutionary history with PPW-1, they only bind sRNAs for a reduced set of TE families with detectable sRNAs (15.7% and 16.2%, respectively). This observation suggests subfunctionalization of SAGO-1/2, possibly due to differences in tissue localization, since SAGO-1 and SAGO-2 are mainly expressed at the apical membrane of the intestine, while PPW-1 shows enrichment in both the germline and the apical membrane of the intestine (Seroussi et al. 2023).

To gain more insight into the diversity of exWAGO guides, we expanded our analysis to other WAGOs in *C. elegans* (Seroussi et al. 2023), for which we calculated Shannon entropy and Simpson's diversity indices using a comparable set of 43 genomic features, including exons, introns, DNA transposons, LTRs, LINEs, and different ncRNA families (Table S7). We normalized counts using average counts per million (CPM) to account for differences in sequencing depth. In this context, Shannon entropy reflects the evenness in sRNA guide production across different annotation categories, while Simpson's diversity highlights the dominance of specific categories. Highly specialized AGOs such as ALG-1 and PRG-1 show low Shannon entropy (0.004 and 0.4, respectively) and low Simpson diversity (0.001 and 0.12) (Fig. S7), highlighting low diversity and dominance of specific categories. This is expected since ALG-1 has a high selectivity for miRNAs, and PRG-1 for piRNAs. However, Shannon entropy in *C. elegans* WAGOs ranges from 0.3 to 2.1 (mean = 1.38, SD = 0.49), while Simpson's diversity ranges from 0.10 to 0.84 (mean = 0.56, SD = 0.19).

In Strongylida, exWAGO exhibits a significantly higher diversity compared to AGOs in *C. elegans* (Wilcoxon one-tailed rank-sum test, *P*-value < 0.001) in both Shannon (mean = 2.22, SD = 0.11) and Simpson indices (mean = 0.84, SD = 0.02). This comparison, however, could be biased by the number of regions producing sRNA bound to these AGO proteins, which is proportional to genome size. Therefore, to account for these differences, we randomly sub-sampled the data for each genome to match the smallest number of enriched regions (WAGO-10, 2,029 genomic regions), but only considering WAGOs, due to the high selectivity of other AGOs like ALGs or PRG-1. For this, we randomly sampled 100 times from each set of enriched regions (see Methods, Fig. 3b). Even using this reduced set, average Shannon and Simpson indices confirm that exWAGO in Strongylida is significantly more diverse than *C. elegans* WAGOs (Wilcoxon one-tailed rank-sum test, *P*-value < 0.001).

Together, these results suggest that the ancestral exWAGO protein was a versatile argonaute, capable of loading sRNAs derived from a wide variety of sources, including most transposon families present in the ancestral genome as well as a subset of protein-coding and non-coding transcripts. This hypothesis is supported by the conserved capacity of PPW-1 to load TE-derived sRNAs, despite the comparatively reduced diversity (likely due to specialization) of its paralogs SAGO-1 and SAGO-2. The strong relationship between exWAGO and TE-derived sRNAs, coupled with its diversity in Strongylida, raises the possibility that this AGO protein has been critical for regulating genome stability in species with high transposable element (TE) activity, ultimately shaping genome architecture over a broad evolutionary timescale.

### ExWAGO as an Ancestral Regulator of LTR Retrotransposons

**Fig. 4.**
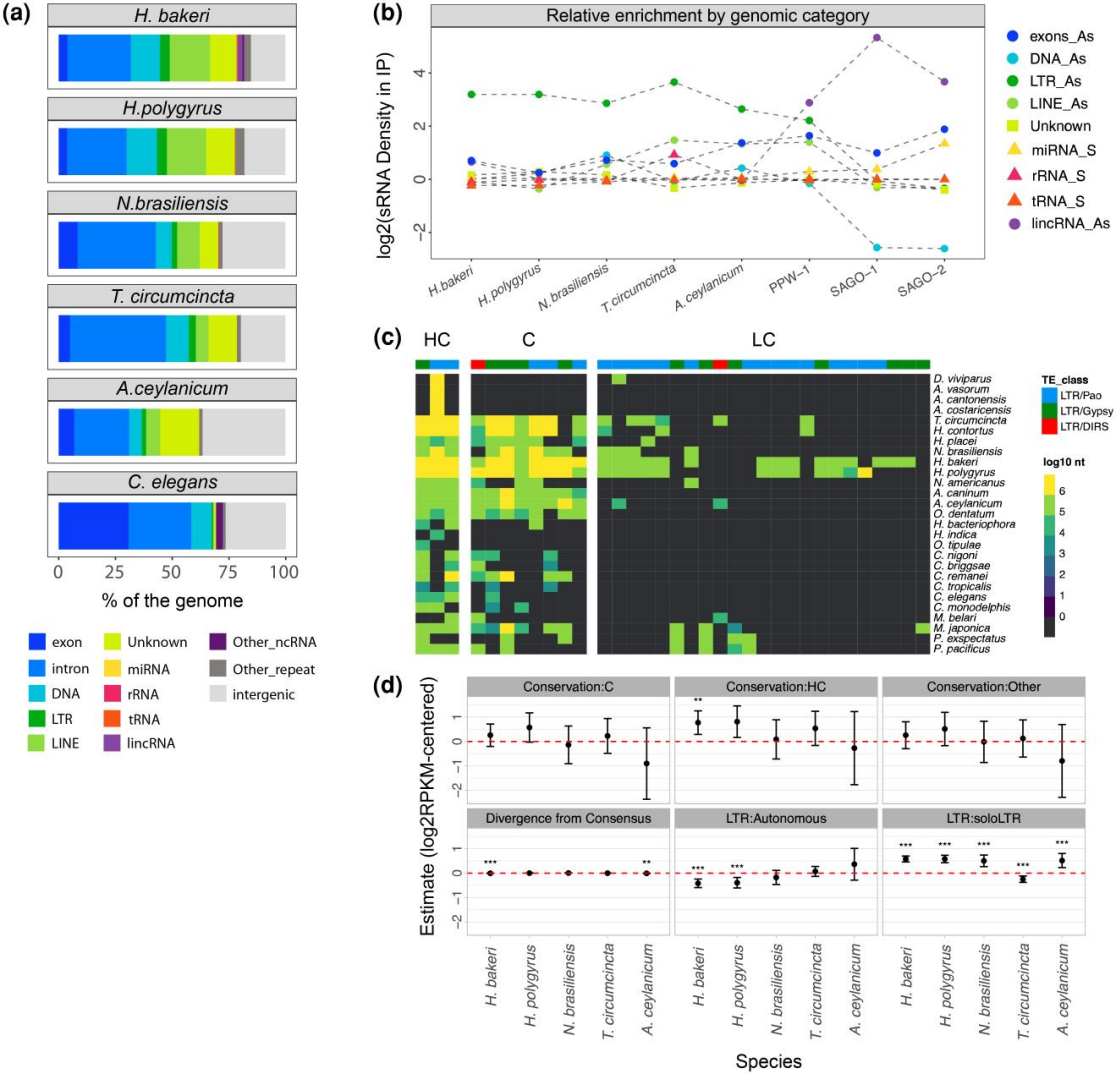
LTR enrichment in exWAGO guide production. a) Comparison of genome annotations in Strongylida parasites and *C. elegans*. b) Comparison of sRNA density of genomic regions enriched in exWAGO guide production per genomic category. The X-axis represents the species, while the Y-axis shows density values estimated as the log_2_ of the ratio of the percentage of CPM of IP-enriched regions and the percentage of genomic bases annotated for each category. c) Phylogenetic orthology inference analysis of LTRs based on the retrotranscriptase (RT) protein. HC = Highly conserved (≥15 species), C = Conserved (5 to 14 species), LC = Lowly conserved (<5 species). The top heat bar indicates LTR classification at the superfamily level. The heatmap shows the genomic abundance of the LTR in log_10_ of bases per HOG. d) Estimates for the fixed effects from the generalized linear mixed model. The red dashed line indicates 0 or a neutral effect (* *P*-value < 0.05, ** *P*-value < 0.01 and *** *P*-value < 0.001).

Although exWAGO binds to a highly diverse set of sRNAs in all parasites, this does not rule out a preference for certain sRNA classes over others. To account for the effect of differences in genome size and annotation coverage, we calculated the density of sRNA for each annotation category (see Methods). Although LTRs are not the most abundant repeats (Fig. 4a), antisense LTR-derived sRNAs show the highest density across Strongylida with 6.2 to 12.6-fold enrichment relative to their genomic span (Fig. 4b). Antisense exon-derived sRNAs are the second-highest category, with an average 3.2-fold enrichment in parasites. In contrast, PPW-1 in *C. elegans* shows a preference for antisense lincRNA-derived sRNAs (7.3-fold enrichment), followed by antisense LTR-derived sRNAs (4.6-fold enrichment), with antisense exon-derived sRNAs in third place (3.1-fold enrichment). Interestingly, when antisense pseudogene-derived sRNAs are taken into account (a category we only have for *C. elegans*), they show a similar enrichment as antisense LTR-derived sRNAs (4.8-fold enrichment) (Fig. S8). Consistent with previous reports (Seroussi et al. 2023), SAGO-1 and SAGO-2 show a strong preference for antisense lincRNA-derived sRNAs, with a 40.3- and 12.7-fold enrichment, respectively, suggesting the function of these orthologs has diverged from that of PPW-1.

Certain genomic categories, especially lincRNAs and pseudogenes, are not easy to annotate de novo, leading them to be underrepresented or absent in genomes other than *C. elegans.* Nevertheless, our results suggest that the common ancestral function of exWAGO was likely related to LTR retrotransposon regulation, while some protein-coding genes also appear to be regulated in all species. This finding is consistent with previous observations in *H. bakeri*, indicating that the most abundant population of antisense siRNAs at the adult stage is retrotransposon-derived sRNAs and protein-coding-derived sRNAs (Chow et al. 2019). The consistent enrichment of LTR retrotransposon-derived sRNA guides across all species raises the question of what drives this conserved association. Is it driven by highly conserved LTR families present in all these species? Are the responsible LTR retrotransposons complete and possibly still active?

### Conserved or Complete LTR Retrotransposons are not the Main Drivers of Extant exWAGO Guide Production

While some of the TE families involved in exWAGO guide production may be conserved across species, their capacity for transposition and degree of sequence divergence may also influence the levels of produced sRNAs. To assess the influence of evolutionary conservation on sRNA levels, we first annotated repetitive elements in 21 additional Strongylida and Rhabditina species encoding exWAGO homologs, using automatically curated libraries (Fig. S9). As observed in *H. bakeri*, *H. polygyrus*, *N. brasiliensis*, *T. circumcincta*, *A. ceylanicum,* and *C. elegans*, LTR retrotransposons are one of the repeat categories with the smallest genomic span across clade V nematodes. To determine their level of conservation, we annotated LTR-encoded protein domains and inferred orthology relationships across all identified families using TEsorter (along with HMM models from Rexdb) and OrthoFinder. Using predicted reverse transcriptase (RT) sequences, we defined phylogenetic hierarchical orthogroups (HOGs) and classified LTR families into 4 categories: highly conserved (present in ≥15 species), conserved (5 to 14 species), lowly conserved (<5 species), and undetermined (lacking a predicted RT) (Fig. 4c). To quantify sequence divergence, we calculated the relative distance between each LTR copy and its consensus using RepeatMasker. To assess completeness, we classified LTR retrotransposons into 3 categories based on coding capacity: autonomous (encoding all transposition-related protein domains), nonautonomous (missing some domains), and soloLTRs (lacking internal coding regions). Finally, to account for differences in element size due to coding capacity or divergence, we normalized sRNA abundance by copy length and library size using reads per kilobase per million (RPKM).

Having classified LTR retrotransposons by conservation and completeness, and estimated their sequence divergence, we fitted a generalized linear mixed model. We used conservation (based on HOGs) and completeness as categorical fixed effects, while divergence as a continuous fixed effect. To account for family-specific sources of variation, we used family as a random effect. Although we initially fitted strand as a random effect, diagnostic plots show that this variable introduces systematic bias into the model, suggesting a confounding effect. Since there is a strong bias toward antisense sRNAs among exWAGO guides (Fig. S6), we used the log_2_ mean-centered RPKM of antisense LTRs as the response variable.

After assessing model fit across species—finding no major deviations from assumptions (Fig. S10)—we observed that, on average, the strongest and most consistent positive effect on sRNA production was associated with soloLTRs (relative to non-autonomous; mean *β* = 0.42 and SE = 0.09) (Fig. 4d and Table S8). In contrast, complete LTR retrotransposons only showed a positive but non-significant effect in *A. ceylanicum* (*β* = 0.35, SE = 0.33, *P*-value = 0.27). Notably, the preference for soloLTR-derived sRNAs remains consistent even when compared separately to either the internal region or the LTR region of full-length LTR retrotransposons (Fig. S11). Meanwhile, high conservation shows a positive effect on sRNA production in some species (compared to lowly conserved; *β* = 0.39, SE = 0.42, on average); however, there is greater uncertainty around this estimate, and in general, the effect is not significant. Finally, the effect of divergence seems to be modest (*β* = −0.003, SE = 0.002, on average) and negatively related to sRNA production, suggesting that less divergent copies (more recent insertions) produce slightly more sRNAs.

Together, our results suggest that the ancestral function of exWAGO in clade V nematodes was related to LTR retrotransposon regulation. Furthermore, despite being non autonomous, soloLTRs are relevant for extant exWAGO guide production, suggesting that retaining these genomic remnants may represent an adaptive advantage either for enhancing TE regulation in trans by silencing active elements of the same family and/or for potential alternative functions of exWAGO.

### Properties of LTRs Associated With sRNA Secretion Pathways

We recently showed that exWAGO can be secreted by *H. bakeri* in 2 forms (inside and outside of EVs) and that exWAGO is internalized by host cells in vivo (Neophytou et al. 2026). Given that EVs containing exWAGO can induce host immunosuppression (Buck et al. 2014), and non-vesicular exWAGO can also enter host cells (Neophytou et al. 2026), we sought to identify the features of LTR retrotransposons that influence the secretion of exWAGO-bound sRNAs. To tackle this, we used vesicular and non-vesicular exWAGO IP libraries from secreted materials.

**Fig. 5.**
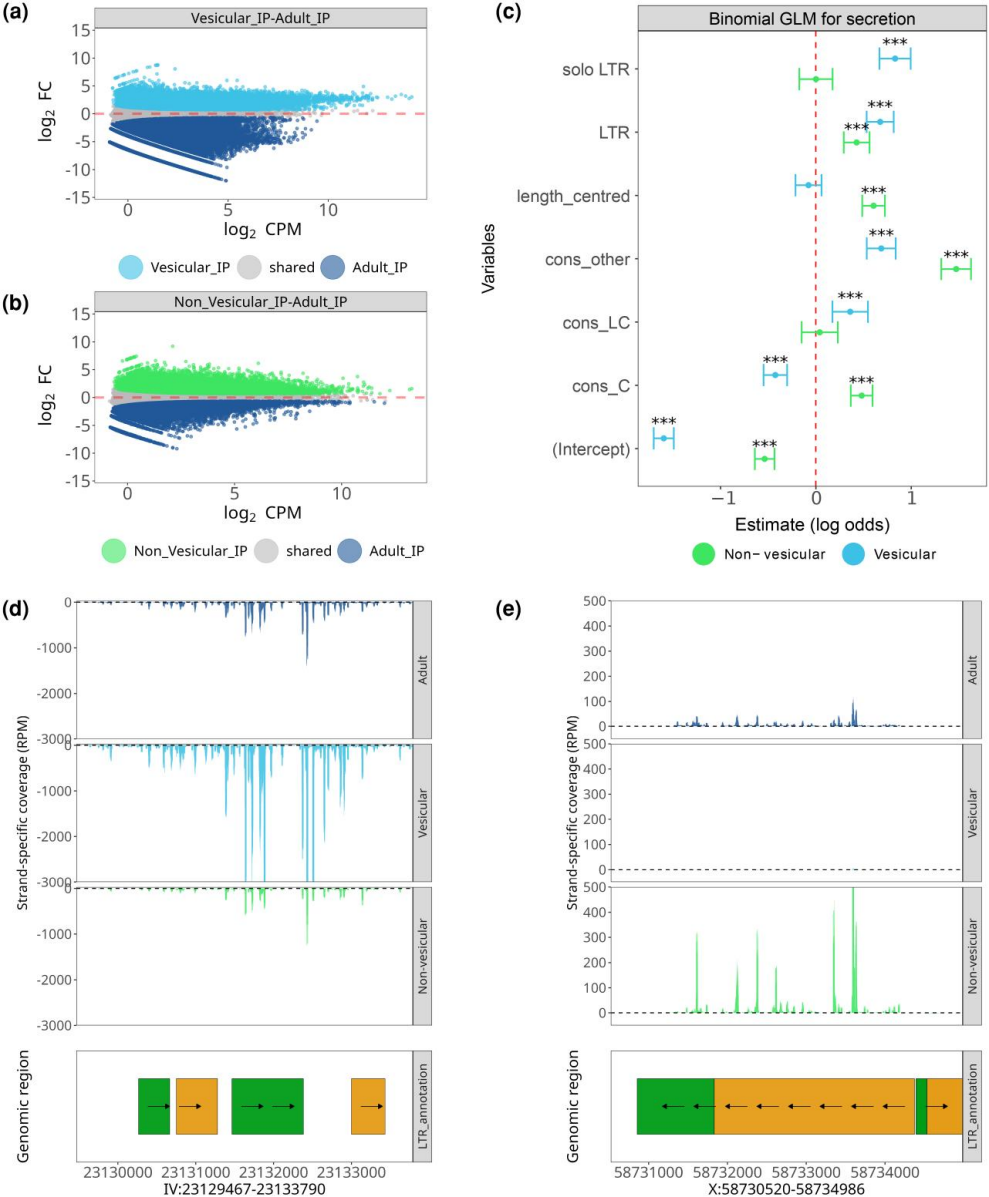
Vesicular and non-vesicular exWAGO guides derive from different types of non-autonomous LTR retrotransposons. a) MA plots (logCPM vs. logFC) after differential expression analysis (FDR < 0.01) of vesicular exWAGO IP against exWAGO IP in adult worms. b) As in (a), but for non-vesicular exWAGO IP against Adult IP. The dashed line indicates log_2_FC = zero. c) Estimated coefficients for binomial generalized linear models testing exWAGO LTR-derived sRNA secretion in vesicular and non-vesicular forms. The dashed line indicates a neutral effect (*** *P*-value < 0.001). d) Examples of a vesicular enriched region showing sRNA production in Adult exWAGO IP, vesicular exWAGO IP and non-vesicular exWAGO IP. e) As in (d) but for a non-vesicular enriched region. Genomic region tracks show LTR retrotransposon annotation (green for the LTR region, orange for the internal coding region).

We performed differential expression analyses to compare the vesicular or non-vesicular exWAGO-IP libraries against the adult exWAGO-IP libraries (see Methods). Using this approach, we identified a set of 18,566 and 24,032 genomic regions enriched in vesicular and non-vesicular exWAGO, respectively (Fig. 5a and 5b).

To determine if LTR retrotransposons involved in secreted (vesicular or non-vesicular) exWAGO guide production differ from those that produce sRNA guides that remain inside the worm, we fitted binomial generalized linear models using the preference for secreted or retained exWAGO guide production as the response variable, encoding regions enriched in secreted exWAGO as 1 and all others as 0. As explanatory variables, we used our previously defined levels of family conservation and completeness. Moreover, we assessed length (centered on the average log_10_ length) to account for differences between different types of LTR retrotransposons. There were no significant deviations from model assumptions in simulated residuals for either model (Fig. S12).

The model results, for the vesicular exWAGO form, indicate that the odds of sRNA secretion increase on average 2.29 times when sRNAs are produced from soloLTR (*β*=0.83, SE = 0.08) and 1.92 from the LTR region (*β*=0.67, SE = 0.07), relative to the internal coding region of LTR retrotransposons, while holding the rest of the variables constant at their base levels (*P* < 0.001) (Fig. 5c and Table S9). Regarding conservation, we found that the odds of sRNA secretion from lowly conserved families (*β*=0.36, SE = 0.09) or undetermined conservation (*β*=0.68, SE = 0.07) due to the lack of detectable RT, increase on average 1.43 and 1.98 times, respectively, compared to highly conserved families (*P* < 0.01). Notably, the mean-centered log_10_ length has a nonsignificant and negative effect (*β*=−0.07, SE = 0.06, *P*-value < 0.26), supporting the preference for localized short regions of LTR retrotransposons, such as the LTR region or soloLTRs, as sources for vesicular-exWAGO guide production.

Our results for the nonvesicular form show that the odds of secretion increase 1.53 times when sRNAs are produced from LTR regions (*β* = 0.43, SE = 0.07, *P* < 0.001). Nevertheless, this preference is not from soloLTRs as with vesicles, but from longer albeit incomplete copies (Fig. 5c and Table S10). Regarding conservation, families annotated as conserved (*β* = 0.48, SE = 0.06) and undetermined (*β* = 1.47, SE = 0.08) are 1.62 and 4.35 times more likely (*P* < 0.001 in both cases), compared to highly conserved families, whereas lowly conserved families show no significant association. Unexpectedly, we also found that length is positively associated with non-vesicular secretion (*β* = 0.60, SE = 0.06), corresponding to a 1.86-fold increase in odds for every 10-fold increase in element length.

Overall, our results indicate that while vesicular exWAGO prefers sRNAs from soloLTRs, the non-vesicular form associates sRNAs from longer incomplete (also non-autonomous) elements. Consistently, both vesicular and non-vesicular exWAGO are associated with LTR families with undetermined conservation (cons_other), due to the lack of RT (Fig. 5c). Therefore, the lack of autonomy for retrotransposition increases the odds for serving as a source for secreted exWAGO guides.

To exemplify regions which produce significantly more secreted exWAGO guides, we highlight a soloLTR on chromosome IV with higher sRNA production (on average 14.44 log_2_ CPM) for the vesicular form (Fig. 5d), and a fragmented LTR retrotransposon on chromosome X (on average 11.8 log_2_ CPM) for the non-vesicular form (Fig. 5e). Both regions show clear differences in our LTR classification, which are consistent with the overall preferences we found using binomial generalized linear models, with the vesicular form showing a preference for soloLTR-derived sRNAs, and the non-vesicular form for longer but incomplete elements.

Collectively, our results delineate 2 selective routes for the source of secreted exWAGO guides: vesicles enriched in lowly conserved, and degenerate soloLTRs, while the nonvesicular form prefers longer but still incomplete elements, with intermediate conservation. Notably, in both secreted exWAGO forms, LTR retrotransposon-derived sRNAs are preferentially produced from nonautonomous elements. This is consistent with the hypothesis that once LTR retrotransposons become inactive, they can remain in the genome and be co-opted for other functions through the production of secreted sRNAs.

## Discussion

To mitigate the potential deleterious effects of TE activity, organisms have adapted molecular components, including argonaute proteins and their sRNA guides to recognize and silence TEs. Most of what we know about TE regulation in nematodes stems from their detailed annotation and functional characterization in *C. elegans*; however, much less is known about these elements and their regulation in parasitic nematodes.

Here, using manually curated TE annotations in multiple Strongylida parasites, we characterize the sRNA preferences of the extracellular worm argonaute exWAGO in an evolutionary context. Our improved TE annotations, combined with refined differential expression analysis, revealed exWAGO as a highly versatile AGO, but with an ancestral function linked to LTR retrotransposon regulation. Although exWAGO guides in adult Strongylida parasites preferentially originate from non-autonomous soloLTRs, the conservation of their corresponding TE family does not appear to influence the levels of sRNAs produced.

Interestingly, exWAGO preference for soloLTR-derived sRNAs extends to extracellular vesicles (EVs) in *H. bakeri*. However, secreted nonvesicular exWAGO shows a particular preference for sRNAs derived from longer and less degenerate elements. These differences related to the 2 secreted exWAGO forms help shape the composition of sRNAs delivered to the parasite's host. The recurrent lack of detectable reverse transcriptase—and therefore retrotransposition autonomy—of these elements indicates a consistent pattern of decay before they become a preferred source of secreted sRNAs.

### Addressing Compositional Bias in Argonaute IPs: rRNA-Guided Normalization

In nematodes, worm-specific argonautes (WAGOs) load sRNAs with specific properties, typically ∼22 nt in length starting with a 5′ triphosphorylated Guanosine (22G sRNAs). Immunoprecipitation and sequencing of bound sRNAs for AGOs that associate with sRNAs from a broad set of loci, such as CSR-1 or WAGO-4 in *C. elegans,* or exWAGO in Strongylida parasites, presents certain challenges. Standard normalization methods such as TMM rely on the assumption that most loci are not differentially expressed (Robinson and Oshlack 2010). This assumption holds in experimental designs where only a subset of genes is expected to change. However, it has been previously shown that when most of the genes in a sample are differentially expressed, this assumption is invalid (Bullard et al. 2010; Zhou et al. 2017), for example, in RNA-seq experiments comparing tissues or development stages with clear differences in transcriptional activity.

Here, by estimating normalization factors based on the adjusted expression of rRNA fragments, we were able to improve the detection of genomic regions enriched in exWAGO guide production. We used rRNA because, in general, we do not expect WAGOs to associate with this type of sRNA (Zagoskin et al. 2022; Seroussi et al. 2023). Although previous reports in humans and mice have shown that rRNA fragments can be enriched in AGOs (Guan and Grigoriev 2021), neither of our analyses in *C. elegans* WAGOs nor exWAGO show general patterns of rRNA enrichment in IPs. Other elements could be used for normalization, for example, miRNAs could be employed for WAGOs that do not bind them. However, using rRNA allows the same approach to be used across all AGOs. This supports the use of rRNA-derived sRNAs as a stable internal reference for normalization in this context.

Although this approach reduces the false negative rate introduced by standard TMM, we caution that this strategy is not perfect, since different amounts of rRNA fragments could originate in samples with different levels of degradation or with higher background levels. Careful inspection of the resulting MA plot is still recommended to verify that the effect of normalization agrees with biological assumptions. In most of our samples, this normalization worked well, but our analysis in *T. circumcincta* indicates higher levels of rRNA fragments in the IP, potentially causing an underestimation of enriched regions, leading to more conservative results than for other species.

In future work, we could consider using controlled spike-ins in both IP and input samples to evaluate alternative normalization strategies. Nevertheless, these problems do not undermine the broader applicability of our normalization strategy, nor the robustness of our biological conclusions, which are supported by consistent patterns of TE enrichment across Strongylida parasites.

### The Dual Role of exWAGO and soloLTR-Derived sRNAs in Strongylida Parasites

Despite their relatively low genomic span in clade V nematodes, LTR retrotransposons have been linked to key processes such as genome expansion, innate immune responses, and adaptation to environmental stress (Ni et al. 2016; Kanzaki et al. 2018; Ansaloni et al. 2019). In *C. elegans*, the argonaute protein HRDE-1 has been implicated in LTR regulation under heat shock conditions (Ni et al. 2016), and ERGO-1 has also been shown to target LTR elements (Fischer and Ruvkun 2020). Notably, the loss of ERGO-1 in *Caenorhabditis inopinata* correlates with a marked expansion of LTR retrotransposons in that species (Kanzaki et al. 2018). Remote homology searches across predicted proteomes of Strongylida parasites revealed that ERGO-1 is absent (Table S11), and our phylogenetic analyses highlight that these species do not have several additional AGO duplications observed in *C. elegans*, while also possibly having lost ERGO-1 (Table S12). We also found that exWAGO exhibits higher sRNA guide diversity relative to *C. elegans* AGOs, which may be a consequence of an even more expanded WAGO repertoire in *C. elegans*, probably leading to further specialization (see Fig. S13). While we cannot exclude the possibility that other argonautes in Strongylida bind LTR-derived sRNAs, our enrichment analyses demonstrate that exWAGO orthologs—from *C. elegans* PPW-1 to parasitic Strongylida species—consistently associate with LTR-derived small RNAs. This suggests that exWAGO retains an ancestral role in LTR retrotransposon surveillance.

But why does exWAGO show this remarkable preference for LTR-derived sRNAs in Strongylida parasites? An increased activity of LTR retrotransposons can lead to genomic instability. In this regard, the preference for soloLTR-derived sRNAs in adult worms may represent an advantage, because by retaining transcriptional activity, they can act as a safe source for sRNAs to potentially regulate full-length elements of the same family. Although not identical, similar mechanisms are known in *Drosophila* species where genomic loci like the flamenco cluster retain soloLTRs that act as the source of piRNAs (Van Lopik et al. 2023), which in turn can modulate active LTR retrotransposons in trans. These parallels raise the possibility that nematodes may have independently selected decayed LTR retrotransposons as safe templates of sRNA biogenesis for TE regulation.

An alternative, nonmutually exclusive possibility is that exWAGO does not solely act as a repressive factor but may also participate in licensing or fine-tuning LTR retrotransposon activity. Such a function has been previously reported for the *C. elegans* WAGO CSR-1 (Wedeles et al. 2013). In this scenario, exWAGO-associated sRNAs could contribute to context-dependent regulation, allowing limited LTR expression, in specific cell-types, and/or under specific developmental or environmental conditions. This fine tuning may provide an advantage, to license TEs associated with secretion, for instance, while preventing a general burst in TE activity. Discriminating between these models will require further functional assays to determine whether exWAGO-bound LTR-derived sRNAs mediate transcriptional silencing, post-transcriptional repression, or conditional activation of LTR retrotransposons.

In addition, the immunogenic capacity we observed for *H. bakeri* EVs and the non-vesicular exWAGO (Buck et al. 2014; Neophytou et al. 2026), hints at a potential role of LTR-derived sRNAs in modulating host immunity. In this context, the preferential export of sRNAs derived from non-autonomous LTR elements may represent a mechanism by which parasites exploit genomic fossils or degenerate elements to mediate cross-species communication. Notably, although we observed that elements of undetermined conservation are consistently preferred for secretion, one limitation of our analysis is that conservation was defined based solely on the reverse transcriptase protein. Our results are consistent in that both secreted exWAGO forms show a preference for non-autonomous LTR elements. We hypothesize that these genomic fossils provide a stable, low-risk source of guides that can be safely exported without fueling retrotransposition. Further work to identify host targets and test functional consequences of these secretion pathways will clarify how widespread and adaptive this strategy is for nematode parasitism.

An important open question is why nematodes such as *H. bakeri* use distinct repertoires of LTR retrotransposons as sources of secreted sRNAs in vesicular versus nonvesicular exWAGO. We previously showed that *H. bakeri* EVs are likely of intestinal origin (Buck et al. 2014), suggesting that vesicular exWAGO-sRNA complexes may predominantly reflect the sRNA population of intestinal cells. Nonetheless, we recently found that exWAGO is broadly expressed in *H. bakeri* (Neophytou et al. 2026), raising the possibility that non-vesicular exWAGO-sRNA complexes are derived from a wider variety of cell types. Based on these observations, we hypothesize that both exWAGO forms are derived from different, although overlapping, cellular origins, thereby sampling different pools of LTR retrotransposon-derived sRNAs.

Intriguingly, beyond parasitic nematodes, fungal pathogens such as *Botrytis cinerea* also rely on LTR retrotransposon-derived sRNAs as a source of extracellular RNA with immunomodulatory potential (Porquier et al. 2021). While *B. cinerea* preferentially uses full-length LTR retrotransposons as the source of secreted RNAs, nematodes like *H. bakeri* show a preference for non-autonomous LTR retrotransposons. This contrast suggests that the evolutionary paths and molecular strategies shaping extracellular RNA repertoires may differ across kingdoms, but highlight a potential convergent source of sRNAs for extracellular RNA communication.

Together, our results support a model in which exWAGO maintains genome integrity by broadly regulating TEs, while also existing extracellularly and carrying distinct sRNA guides. This dual role positions exWAGO at the interface of genome defense and parasite-host communication, suggesting the possibility that certain LTR retrotransposons have been, or are in the process of being, co-opted for extracellular signaling in parasitic nematodes.

## Methods

### Transposable Element Annotation

We produced de novo repeat annotations using earlGrey v.4.0.3 (Baril et al. 2024), with the Nematoda Dfam library (Dfam 3.7), and 10 iterations of the “BLAST, Extract, Extend” process. For *H. bakeri* and *H. polygyrus*, we used our previously published curated libraries (Stevens et al. 2023), with some minor updates.

Redundancy within the predicted libraries was resolved by collapsing models with at least 80 nt length and sharing 80% of nt identity in at least 80% of the model to the centroid model (the longest one), using cd-hit-est (Li and Godzik 2006). To remove non-TE-related proteins, getorf from the EMBOSS package was implemented to obtain ORFs with a minimum size of 300 aa, to subsequently annotate protein domains using PfamScan (http://ftp.ebi.ac.uk/pub/databases/Pfam/Tools/) with the Pfam database (RELEASE 35). Models with hits to non-TE-related proteins were filtered out. Furthermore, TEsorter 1.4.6 was used to identify TE-related protein domains (Zhang et al. 2022). Unknown models, with hits for RT, ENDO, and INT, were further classified as LINE or LTR based on TEsorter results. Moreover, TE-Aid (available at https://github.com/clemgoub/TE-Aid) was used to identify the structural domains of predicted models. We classified them as DNA/MITE models when showing only TIR structures in self-dot plots and less than 1 kb (Goubert et al. 2022).

To have insights into the evolution of transposable elements in nematodes where exWAGO can be secreted, we manually curated the models of *N. brasiliensis* and *T. circumcincta*. For *A. ceylanicum*, we only manually curated LTR retrotransposons and the models required to annotate 80% of the total repeat content. For this, we retrieved up to 50 instances of each element, extending 1,000 nt of each edge, using the make_fasta_from_blast.sh (with the parameters 0 and 1,000) and ready_for_MSA.sh (with the parameters 50 and 40) scripts. (https://github.com/annaprotasio/TE_ManAnnot). The resulting sequences were aligned using mafft v7.520, and the alignments were inspected using AliView v1.28. We identified the canonical TGT and CAC motifs at the beginning of LTRs and TAT for DNA transposons to trim divergent or poorly aligned columns (Goubert et al. 2022). For models where the edges were highly degenerate, we used CIAlign (Tumescheit et al. 2022) with the parameters: –insertion_max_size 50 –crop_ends –crop_divergent –crop_divergent_min_prop_ident .5 –crop_divergent_min_prop_nongap .6 –crop_divergent_buffer_size 15 –make_consensus. Consensus models from the resulting alignments were built using CIAlign with the make_consensus function.

Resulting libraries were then used to produce annotations using RepeatMasker with the parameters (-s -cutoff 400 -a). Since RepeatMasker produces fragmented annotations, the RepeatCraft pipeline with the Loose mode (Wong and Simakov 2019; Baril et al. 2024) from earlGrey was used to collapse fragmented annotations. Overlapping bases between repeats were assigned using the 01.assign_TE_overlaps.R (available at https://github.com/imu93/ms_evo_exwago). Briefly, elements belonging to the same superfamily were collapsed into a single element using the reduce function from the GenomicRanges R package (Lawrence et al. 2013). If overlapping elements belonged to a different family, the family of the collapsed segment was assigned based on the longest element. Moreover, overlapping bases of elements of different super-families were assigned based on a hierarchical classification. These bases were assigned to the element that belongs to the superfamily with more bases in the genome.

### Benchmark of Curated TE Libraries

TE library benchmarking was conducted using the get_family_summary script (available at: https://github.com/jmf422/TE_annotation/blob/master/get_family_summary_paper.sh). This script classifies consensus sequences of a tested library, based on their degree of sequence similarity, coverage, and divergence, relative to a curated library. The classification consists of 4 categories: perfect (>95% of sequence similarity, > 95% of coverage, and <5% of divergence), good (same as perfect but up to <10% of divergence), present (>80% of coverage), and missed (no hits or <80% of coverage).

### NcRNA Gene Annotations

ncRNA annotation was carried out using several homology-based tools. We used infernal v1.1.4 and Rfam v.14.10 covariance models to annotate known ncRNA families (–rfam –cut_ga –nohmmonly). In addition, tRNAscan-SE (-Q -E –score 40) (v1.4) and RNAmmer (-S euk -m tsu,lsu,ssu) (v1.2) were used to annotate tRNAs and rRNAs, respectively. miRNA annotation was conducted using the precursors of miRNAs for nematodes in miRBase (v.22). After obtaining precursors, blastn was used (-evalue 1e-5 -perc_identity 90 -qcov_hsp_perc 90). In addition, miRNA annotation of *N. brasiliensis*, *T. circumcincta,* and *A. ceylanicum* was enhanced by using data from regular sRNA-seq libraries (NCBI Bioproject PRJNA1200757) using MirDeep2 (Friedländer et al. 2012).

Meanwhile, piRNAs were annotated only in a subset of species. The annotation of this ncRNA family was conducted using bowtie1 (-f -a –best –strata -v 0 -m 3 -S) and previously identified type 1 piRNAs in *H. bakeri*, *N. brasiliensis* (Beltran et al. 2019). Given the close phylogenetic relationship between *H. bakeri* and *H. polygyrus*, we used *H. bakeri* as a reference to annotate putative conserved piRNAs in *H. polygyrus*. Using a similar approach, we annotated piRNAs in a chromosome-level assembly of *N. brasiliensis*.

LncRNAs and lincRNAs were annotated using publicly available RNA-seq data in *H. bakeri*, *N. brasiliensis*, *T. circumcincta*, and *A. ceylanicum* (*H. bakeri*: PRJNA750155; *N. brasiliensis*: PRJEB16076, PRJEB20824, SRR25993873; *T. circumcincta*: PRJEB7677; *A. ceylanicum*: PRJNA231490). Briefly, we aligned RNA-seq reads to their respective genome using STAR v.2.7.10b using the gtf gene annotation file to define junctions. The resulting bam files were sorted using samtools sort (Li et al. 2009) to build transcriptomes with StringTie v.2.2.1 (Pertea et al. 2015). We evaluated the transcript coding potential using FEELnc_filter, cpc2, lgc, and PfamScan (Mistry et al. 2021; Kang et al. 2017; Wucher et al. 2017; Wang et al. 2019), to filter out transcripts produced from protein-coding genes. To classify lncRNAs and lincRNAs, FEELnc_classifier was used (Wucher et al. 2017). In addition, lncRNAs and lincRNAs were annotated in *H. polygyrus* using blastn with the predicted lncRNA and lincRNAs in *H. bakeri* as reference (-evalue 1e-10 -perc_identity 90—qcov_hsp_perc 90).

### Segmented Annotations

Aiming to understand the contribution of different genomic categories to sRNA production, we built non-overlapping annotations in which each base in the genome has a single annotation. We assigned overlapping regions based on a hierarchical classification using the setdiff function from the GenomicRanges R package (Lawrence et al. 2013). This annotation includes both sense and antisense annotations for each genomic region. Our hierarchy prioritizes known sRNA families (in the following order: miRNA, piRNA, yRNA, rRNA, tRNA, snRNA, snoRNA, lincRNA, lncRNA), then TEs (TE families were ranked based on their genomic span), exons, introns, and finally, in the last part of the hierarchy we included unstranded repeats such as MITEs, SINEs, Low complexity repeats, Simple repeats, and Unknown repeats, in that order.

### ExWAGO Immunoprecipitations and RNA Isolation From Worms

To interrogate the sRNAs associated with exWAGO in different nematode species, including *H. bakeri*, *T. circumcincta*, *N. brasiliensis,* and *A. ceylanicum* we used previously published sRNA library datasets (NCBI Bioproject PRJNA1200757) that were generated by using the eluate and unbound fraction of exWAGO immunoprecipitation from adult worms.

Here, we generated 2 new sets of libraries for *H. polygyrus.* First, we generated exWAGO-IP sRNA libraries from wild-caught, naturally *H. polygyrus*-infected wood mice (*Apodemus sylvaticus*) from woodlands in Midlothian, Scotland (eg Penicuik Estate (55°49′N 3°15′W) and Hewan Woods (55°52′N 3°08′W). To immunoprecipitate exWAGO from these samples, ∼1.5 cm of the duodenum was harvested and flash frozen immediately in liquid nitrogen to minimize RNA degradation, and stored in −70 °C until required. Protein G beads (ThermoFisher, 10003D) were washed 5 times using cold Binding Wash Buffer (PBS, 0.02% Tween-20) and polyclonal rat anti-exWAGO serum antibodies (Neophytou et al. 2026) were conjugated on the prepared beads by incubation (2 h, rotating wheel, 4 °C). Unconjugated antibody was then removed, and the beads were equilibrated using cold lysis buffer (150 mM NaCl, 10 mM Tris.HCl, 0.5 mM EDTA, 0.5% NP40) containing protease inhibitors (1 tablet per 5 ml) (Roche, 11873580001) 3 times. Then, the frozen tissue was ground into a fine powder using a pestle and mortar under liquid nitrogen and then lysed in 1 ml of cold lysis buffer as above supplemented with 200 U/ml RNase inhibitors (Promega, N2515). Unlysed material was removed by centrifugation (16,100 rcf, 10 min, 4 °C), and the supernatant lysate was incubated with the antibody-conjugated beads (45 min, rotating wheel, 4 °C). The beads were washed with cold Low Salt buffer (50 mM Tris.HCl pH 7.5, 300 mM NaCl, 5 mM MgCl2, 0.5% NP40 and 2.5% glycerol), followed by 2 washes with cold High Salt buffer (50 mM Tris.HCl pH 7.5, 800 mM NaCl, 10 mM MgCl2, 0.5% NP40 and 2.5% glycerol) (5 min, rotating wheel, 4 °C). The beads were then washed once with cold Low Salt buffer, followed by a cold PNK buffer wash (50 mM Tris.HCl pH 7.5, 50 mM NaCl, 10 mM MgCl2, 0.5% NP40). For small RNA sequencing analysis, the RNA was eluted directly in Qiazol (700 μl, 5 min, room temperature) (Qiagen) and stored at −70 °C until required. Secondly, we generated total sRNA libraries from adult *H. polygyrus* worms harvested from lab-reared wood mice at day 14 post-challenge. This outbred colony of wood mice was originally wild-caught, but is now maintained in standard laboratory conditions at the University of Edinburgh (see Sweeny et al. 2021 for more details). Worms were washed in PBS and then lysed in 700 μl of Qiazol by bead beating using 5 mm steel beads (Qiagen, 69989) using the Tissue Lyser II (Qiagen) in pre-cooled cartridges for 2 min at 30 Hz twice. The RNA was then processed for sequencing as described below.

### Small RNA Libraries

sRNA libraries were generated as described in Neophytou et al. (2026). Briefly, RNA in Qiazol was spiked with 7 μl of 10 pM RT4 synthetic spike (CUUGCGCAGAUAGUCGACACGA). The RNA was then extracted using the miRNA Serum/Plasma kit (Qiagen, 217184) and treated with RNA 5′ Polyphosphatase (Lucigen, RP8092H) according to the manufacturer's instructions. The reaction was terminated using ethanol precipitation (−70 °C, overnight), and the precipitated RNA was resuspended in 2.5 μl of nuclease-free water and 2.5 μl of Buffer 1 (TriLink, L-3206). The sRNA libraries were prepared using the CleanTag Small RNA Library Preparation Kit (TriLink, L-3206) using half reaction volumes. The *H. polygyrus* immunoprecipitation or total adult worm samples were generated using 1:10 or 1:12 dilution of 5′ and 3′ adapters, respectively. All libraries were generated using 20 amplification cycles, and the profile of the libraries was assessed using the High Sensitivity DNA Bioanalyser chip (Agilent, 5067-4626). Pooled libraries were size-selected (140 to 180 bp for immunoprecipitation; 140 to 220 bp for total adult worms) using gel purification to remove adapter dimers. The libraries were sequenced on the Illumina NextSeq 2000 platform by the Edinburgh Clinical Research Facility using single-end 100 bp reads.

### Small RNA: Processing, Alignment and Quantification

In addition to the small RNA-seq libraries we produced for *H. polygyrus*, we obtained small RNA-seq libraries from NCBI SRA for *H. bakeri* (NCBI bioprojects: PRJNA481340 and PRJNA1200757)*, N. brasiliensis* (PRJNA1200757), *T. circumcincta* (PRJNA1200757), *A. ceylanicum* (PRJNA1200757), and *C. elegans* (PRJNA860633). Illumina small RNA adapters were removed using reaper (Davis et al. 2013). FastQC and MultiQC were used to evaluate read quality (Andrews, 2010; Ewels et al. 2016). For *T. circumcincta,* we collapsed technical replicates for exWAGO IP libraries; however, due to the lack of biological replicates, technical replicates for adult total libraries were treated as independent samples. A similar approach was used for *A. ceylanicum*. Using Pullseq v.1.0.2 (available at https://github.com/bcthomas/pullseq), we then retrieved reads between 18 and 27 nucleotides for downstream analyses. ShortStack v.3.8.5 (Johnson et al. 2016) was used for genome indexing and alignment with the following parameters: bowtie-buld –offrate 2; –nohp –mincov 5 –pad 1 –mismatches 1 –mmap u –bowtie_m all –ranmax “none” –bowtie_cores 32 –sort_mem 248G. After using samtools split (Li et al. 2009) to obtain individual bam files, the featureCounts function from Rsubread (Liao et al. 2019) was used to quantify aligned sRNAs to segmented annotations (see segmented annotation above) (parameters: allowMultiOverlap = TRUE, strandSpecific = 1, fracOverlap = 0.7, useMetaFeatures = TRUE, largestOverlap = TRUE).

### Differential Expression and Enrichment Analyses of exWAGO and Adult Total Across Clade V Nematodes

We performed individual differential expression analysis comparing exWAGO IP against input libraries (adult total) using edgeR (Robinson et al. 2010). For *C. elegans* we compared PPW-1, SAGO-1, and SAGO-2 IPs against input samples (data from Seroussi et al. 2023). We removed regions with less than 5 CPM in conditions with fewer replicates using the filterByExpr function from edgeR v.4.0.16 (Robinson et al. 2010).

We then estimated normalization factors using the TMM normalization method with the calcNormFactors function. After this, we fitted negative binomial generalized log-linear models using the glmFit function. After visualizing diagnostic MA plots and determining that TMM assumptions are broken in these contrasts, new normalization factors were estimated using the expression of rRNA fragments (those that mapped in sense). To account for compositional bias of rRNA in the different libraries, we used TMM on rRNA genes, reducing the effect of any extreme values for normalization factor estimation. We then used glmFit and glmTreat to fit negative binomial generalized log-linear models and determine enriched regions. Genomic regions that were not enriched in the IPs were considered as unbound.

### Argonaute sRNA Guide Diversity in Strongylida and *C. elegans*

To compare the diversity of sRNA guides between exWAGO orthologs in Strongylida parasites and *C. elegans* argonautes, aiming to account for differences in sequencing depth between IP experiments, we normalized raw counts into counts per million (CPM). We then used the vegan R package to estimate Shannon's and Simpson's diversity indices (Oksanen et al. 2026). For this, we used the average of 100 rarefied random samplings using the number of enriched regions in WAGO-10 (2,029 regions) as the standardized sampling size. Statistical comparison was then carried out using the ggpubr R package (Kassambara 2025).

### LTR Classification, sRNA Quantification and Differential Expression Analysis

To further classify the features of the genomic regions initially annotated as LTR retrotransposons, we first split the curated consensus sequences of LTR retrotransposons into the LTR (directed repeats) and internal region regions (containing CDS of protein domains), using selfblast produced with TE-Aid, and an ad hoc R script (available at: https://github.com/imu93/ms_evo_exwago). Using the split models, we build a new LTR retrotransposon library. After splitting the models, we selected only one LTR region per consensus model. In models where the nucleotide identity was lower than 80% on their directed repeats or lacked directed repeats, we maintain the model, but without this new classification. Using RepeatMasker (-s -cutoff 400 -a -nlow) (Smit et al. 2013), we then reannotate all the genomic segments previously identified as LTR retrotransposons. These new annotations were used to quantify the assigned sRNAs using the featureCounts function from the Rsubread package (strandSpecific = 1, fracOverlap = 1, useMetaFeatures = TRUE, largestOverlap = TRUE) (Liao et al. 2019).

We further performed differential expression analysis using edgeR v.4.0.16 (Robinson et al. 2010). First, we performed differential expression analysis between IP against Unbound for both vesicular and non-vesicular exWAGO, and adult IP against adult total, using housekeeping normalization with rRNA as a reference category. We then fit negative binomial generalized log-linear models with glmFit, and define differentially expressed regions using glmTreat (FDR < 0.05 and log_2_ FC = 1 for secreted exWAGO contrasts and FDR < 0.01 and log_2_ FC = 1 for adult IP against adult total). Finally, using the union of differentially expressed regions, we performed a secreted IP against the adult IP contrast. Since this contrast does not involve the comparison of unbalanced conditions, we used TMM for normalization factor estimation, followed by glmFit and glmTreat (FDR < 0.05 and log_2_ FC = log_2_(1.2)).

### Orthology Inference of LTR Retrotransposons

We performed orthology inference of LTR retrotransposon families among clade V nematodes based on protein identification and phylogenetic reconstruction using Orthofinder v.2.5.4 (Emms and Kelly 2019). For this, we first annotated retrotransposon proteins within each copy using TEsorter (Zhang et al. 2022) with Hidden Markov Models (HMMs) from the metazoan Rex database (Neumann et al. 2019). RexDB was chosen because it provides a curated, lineage-resolved classification of LTR retrotransposons based on conserved protein domains, enabling accurate classification and detection of complete elements. We then used an ad hoc R script to select TE LTR retrotransposon proteins with at least 80% of model coverage and *E*-value < 1e-5.

The orthology of predicted protein domains was interrogated using Orthofinder (Emms and Kelly 2019) to produce phylogenetic hierarchical orthogroups and build phylogenetic trees. For clustering, an inflation value of 6 was used. In addition, a phylogenetic tree based on 443 single-copy ortholog BUSCO genes was used to reconcile gene trees.

### Generalized Linear Mixed Model for LTR-Derived sRNA Production

Using expression values, conservation, divergence, and completeness information from LTR retrotransposons enriched in exWAGO guide production, we fitted a generalized linear mixed model using the glmmTMB R package (McGillycuddy et al. 2025). We estimate log2 reads per kilobase per million (RPKM) values using the rpkm function from edgeR (Robinson et al. 2010), we then mean-centered the values by subtracting the mean log2 RPKM estimated for antisense-derived sRNAs. As fixed categorical effects, we used conservation encoded as highly conserved (HC), conserved (C), lowly conserved (LC), and undetermined. In addition, we modeled LTR type as autonomous (considering the presence of GAG, protease, reverse transcriptase, endonuclease, and integrase domains), non-autonomous (lacking at least one of these 5 protein domains), and soloLTR (based on the LTR region, and lack of coding potential or additional surrounding LTR regions). We also used the RepeatMasker estimated relative divergence values per copy as a continuous fixed effect (Smit et al. 2013). To account for the variance each family has, we also modeled family as a random effect. The same model was independently used for each species, and per-species model fit was assessed using diagnostic qq, residual versus fitted, residual density, and fixed effect distribution plots, with no major deviations from assumptions (Fig. S9).

### Binomial Generalized Linear Model for Secreted exWAGO Guides

To assess which factors influence exWAGO guide secretion from LTR retrotransposons, we fitted a binomial generalized linear model using the lme4 R package (Bates et al. 2015). We fitted enrichment in exWAGO EV IP and non-enrichment as a dichotomic response variable. As explanatory variables, we fitted conservation (based on previously defined phylogenetic hierarchical orthogroups HOGs), strand (sense or antisense), and completeness classification (soloLTR, LTR region, internal-coding region) as categorical. In addition, to account for differences in length, we fitted log10-transformed mean-centered length as continuous. Model assumptions were inspected using the DHARMa R package (Hartig 2024), with no deviations observed in the simulated residuals (Fig. S11).

## Supplementary Material

evag117_Supplementary_Data

## Data Availability

New small RNA-seq libraries for exWAGO IPs and total adults, from *H. polygyrus* are available at the NCBI SRA under BioProject PRJNA1327922. Previously published sRNA-Seq data that were reanalyzed in this manuscript are available under BioProjects PRJNA1200757 and PRJNA481340. Scripts related to differential expression analysis, genome annotation, and repeat libraries are available at https://github.com/imu93/ms_evo_exwago. Curated repeat libraries were also submitted to Dfam. Genome annotation and repeat libraries are also available at https://zenodo.org/records/18235519.
