# Supplementary material for: Comparative Transcriptomics Reveals an Extracellular Worm Argonaute as an Ancestral Regulator of LTR Retrotransposons
Source: Genome Biol Evol. 2026 May 8;18(5):evag117. doi: 10.1093/gbe/evag117 (PMC13195028; doi:10.1093/gbe/evag117)
Supplement: evag117_Supplementary_Data [file evag117_supplementary_data.zip › GBE Supplementary Figures.pdf]

Comparative transcriptomics reveals an extracellular worm Argonaute as an ancestral regulator of LTR retrotransposons

Supplementary Figures

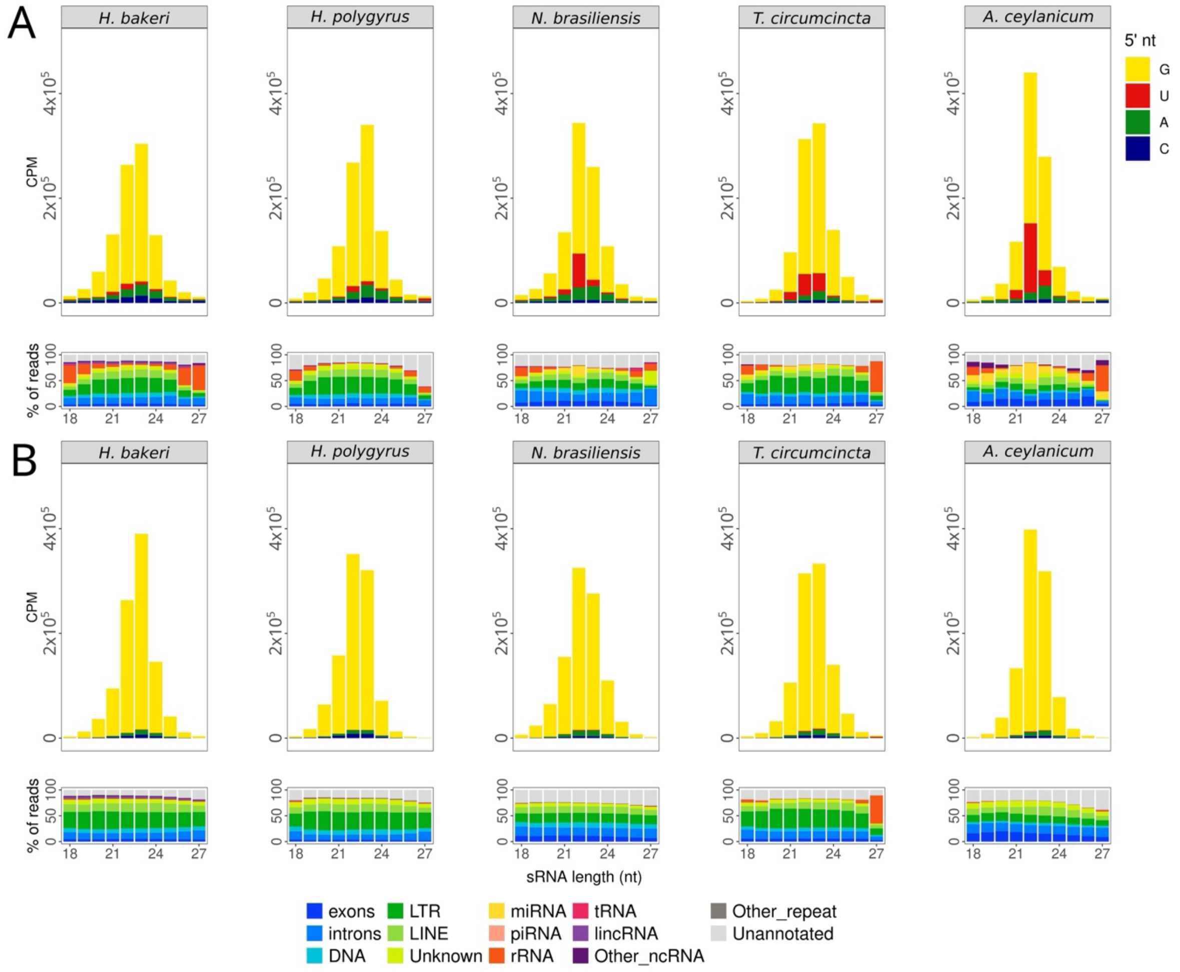

Figure S1. Length, 5′ nucleotide bias, and genomic annotation of small RNAs in Strongylida parasites (*H. bakeri*, *H. polygyrus*, *N. brasiliensis*, *T. circumcincta*, *A. ceylanicum*). (A) Results for Adult-worm (input) libraries. **(B)** Results for exWAGO IP libraries. The top row in both (A) and (B) shows the first nucleotide CPMs (y-axis) of each read divided by length (18-27nt, x-axis). The bottom rows show the percent of all reads, divided by length, mapping to each genomic annotation. All figures represent the sRNA population after 5' polyphosphatase treatment.

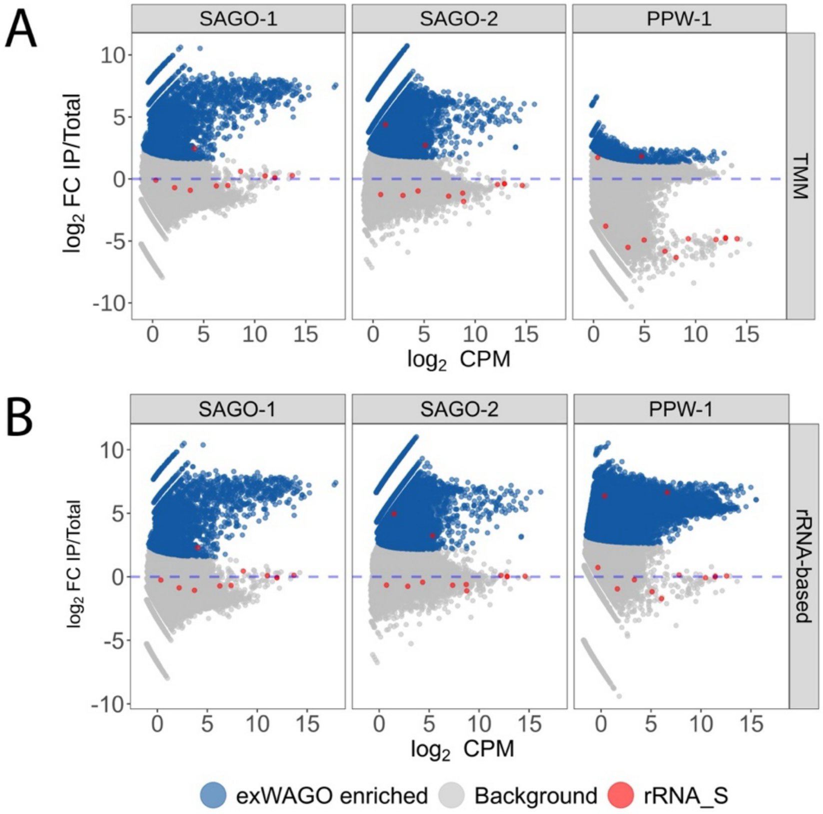

Figure S2. Differential expression analysis for *C. elegans* SAGO-1, SAGO-2 and PPW-1 IPs. (A) MA plots after using TMM normalisation. (B) MA plots after using rRNA-based normalisation. Each dot in the MA plots represents a non-overlapping genomic region. Blue dots highlight regions significantly enriched in IP, with remaining dots in grey, suggesting regions producing mostly unbound sRNAs. Horizontal dotted lines indicate logFC = zero. Red dots indicate the expression of rRNA.

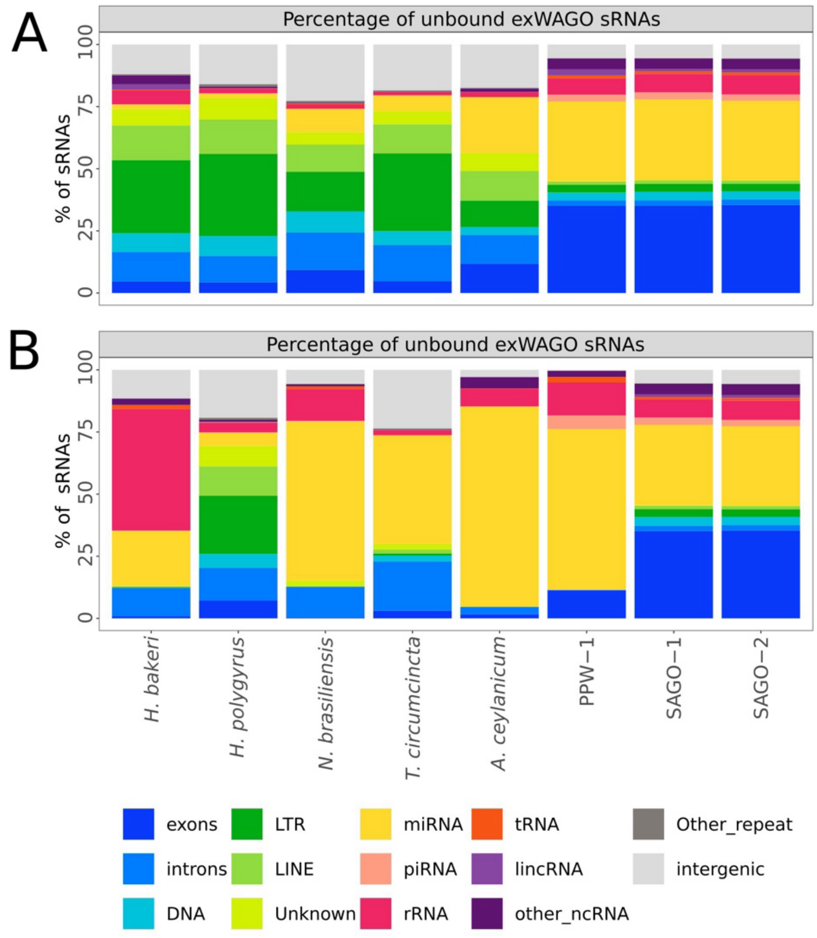

Figure S3. Comparison of sRNAs predicted to remain unbound to exWAGO and its orthologs in Strongylida and *C. elegans*. (A) Results after performing TMM normalisation. (B) Results after performing rRNA-based normalisation. Bars were coloured according to the annotated genomic regions, and represent the percent of reads predicted to remain unbound according to either normalisation method.

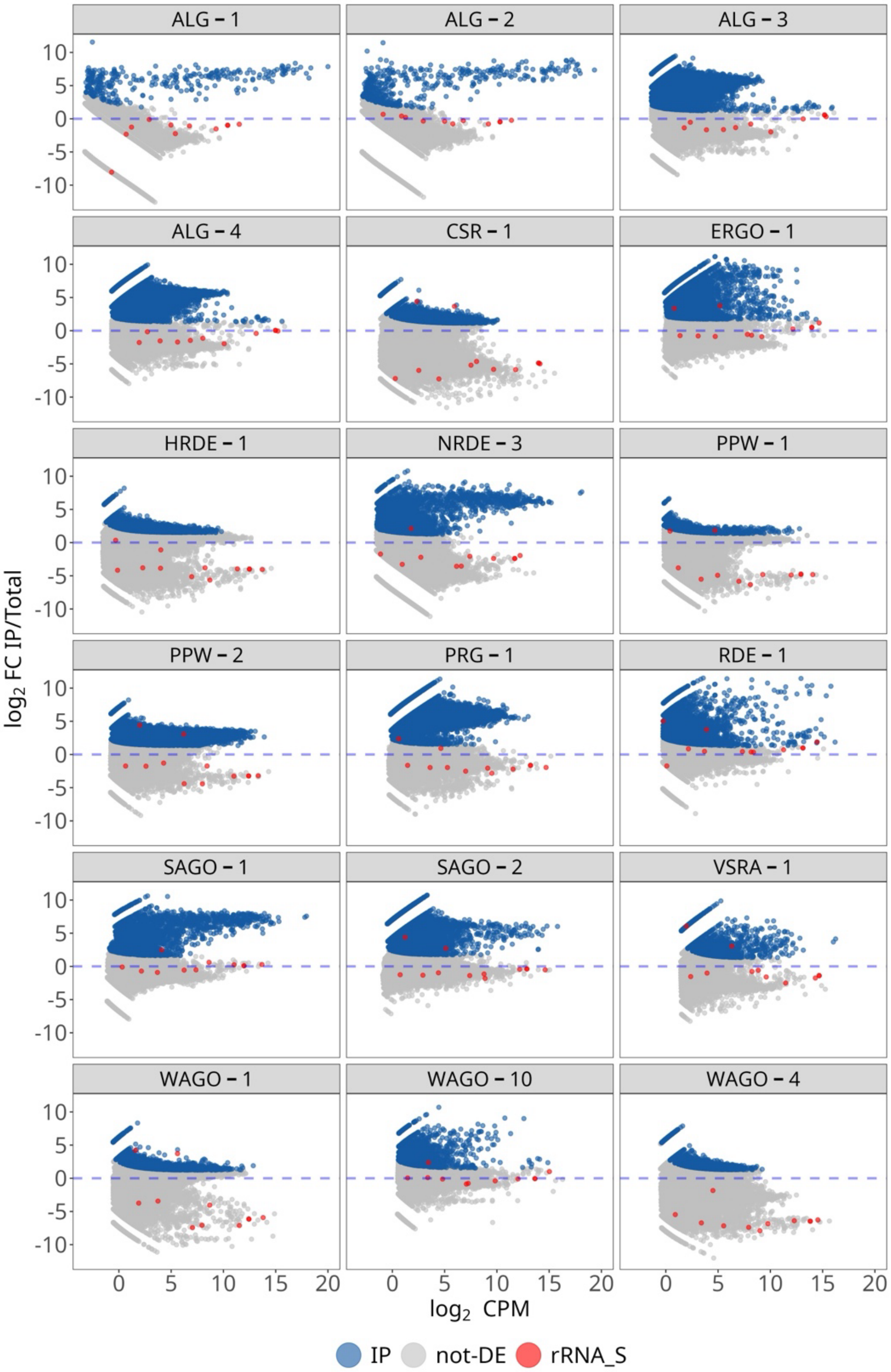

Figure S4. Differential expression analyses using trimmed mean of M-values (TMM) of *C. elegans* AGO IPs against input. Blue dots on each MA plot show the genomic regions enriched in IP (FDR < 0.05), grey dots represent unbound genomic regions, and red dots represent expression values for sense-stranded rRNA fragments (for numerical results see Supplementary Table S5).

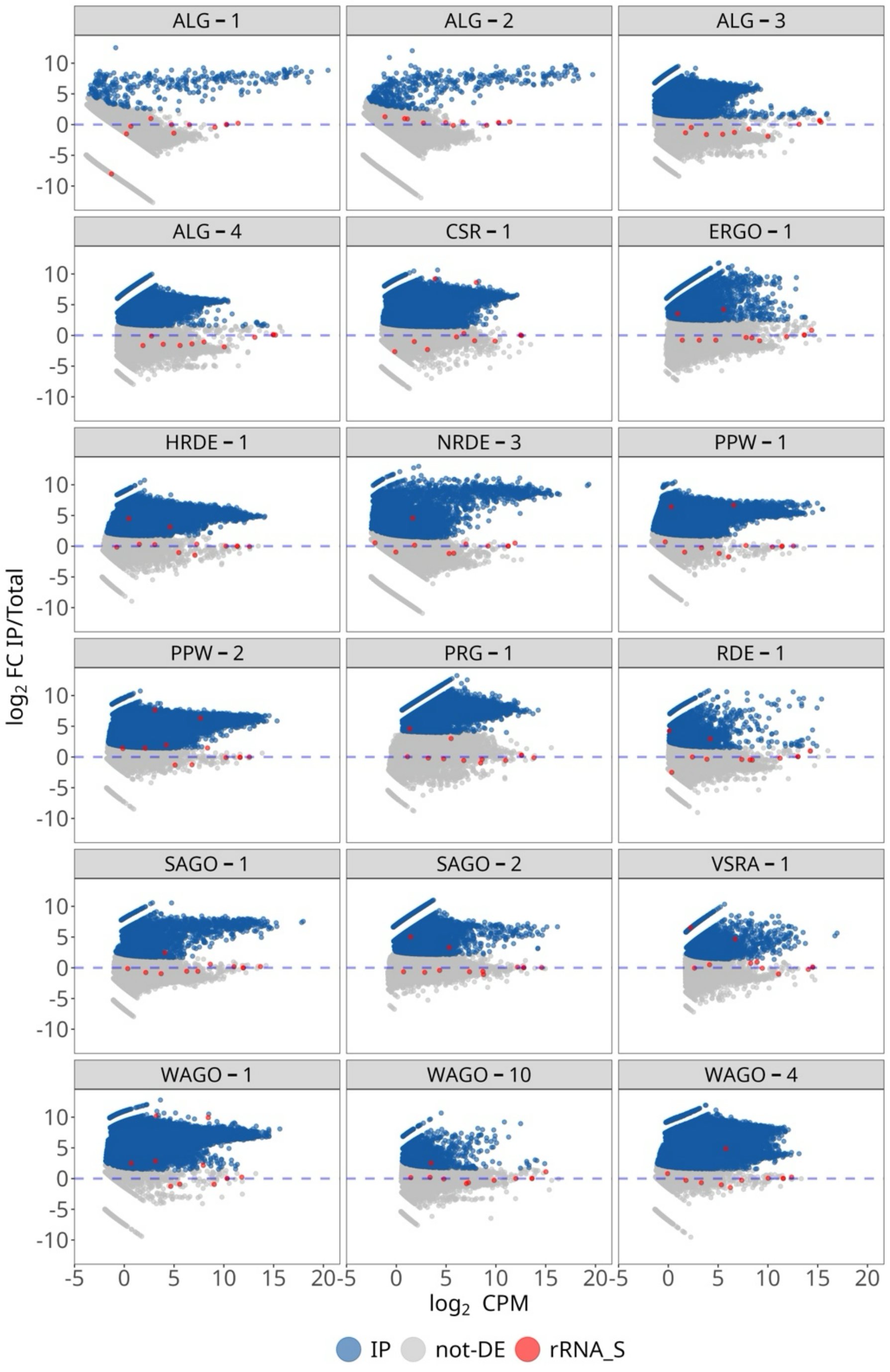

Figure S5. Differential expression analyses using rRNA-based housekeeping normalisation of *C. elegans* AGO IPs against input. Blue dots on each MA plot show the genomic regions enriched in IP (FDR < 0.05), grey dots represent unbound genomic regions, and red dots represent expression values for sense-stranded rRNA fragments (for numerical results see Supplementary Table S5).

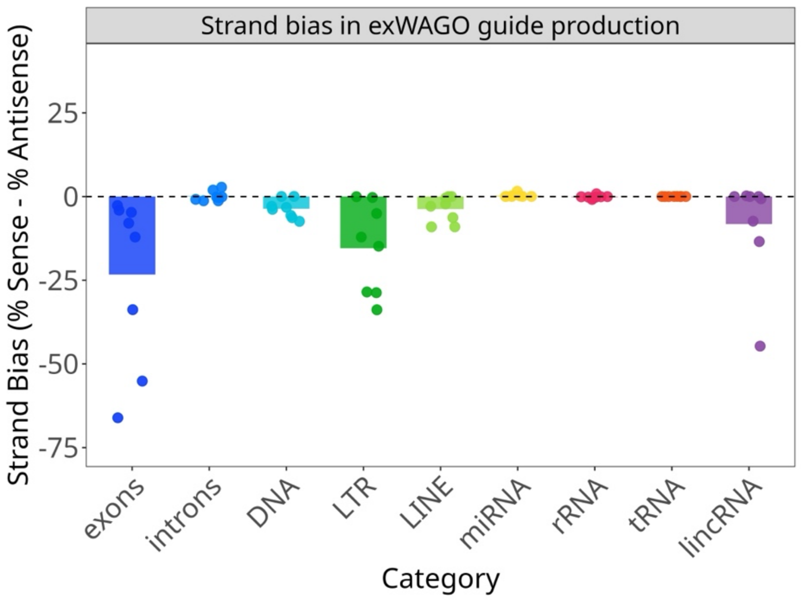

Figure S6. Antisense strand bias in exWAGO and *C. elegans* orthologs guides. Individual dots represent average strand bias values calculated per genomic category and per species, for regions enriched in sRNA guide production. Each bar represents the mean strand bias estimated for all the species. A value of 0 (black dashed line) indicates equal sense and antisense guide production. Positive values indicate a bias toward sense-strand guides, while negative values indicate a bias toward antisense-strand guides.

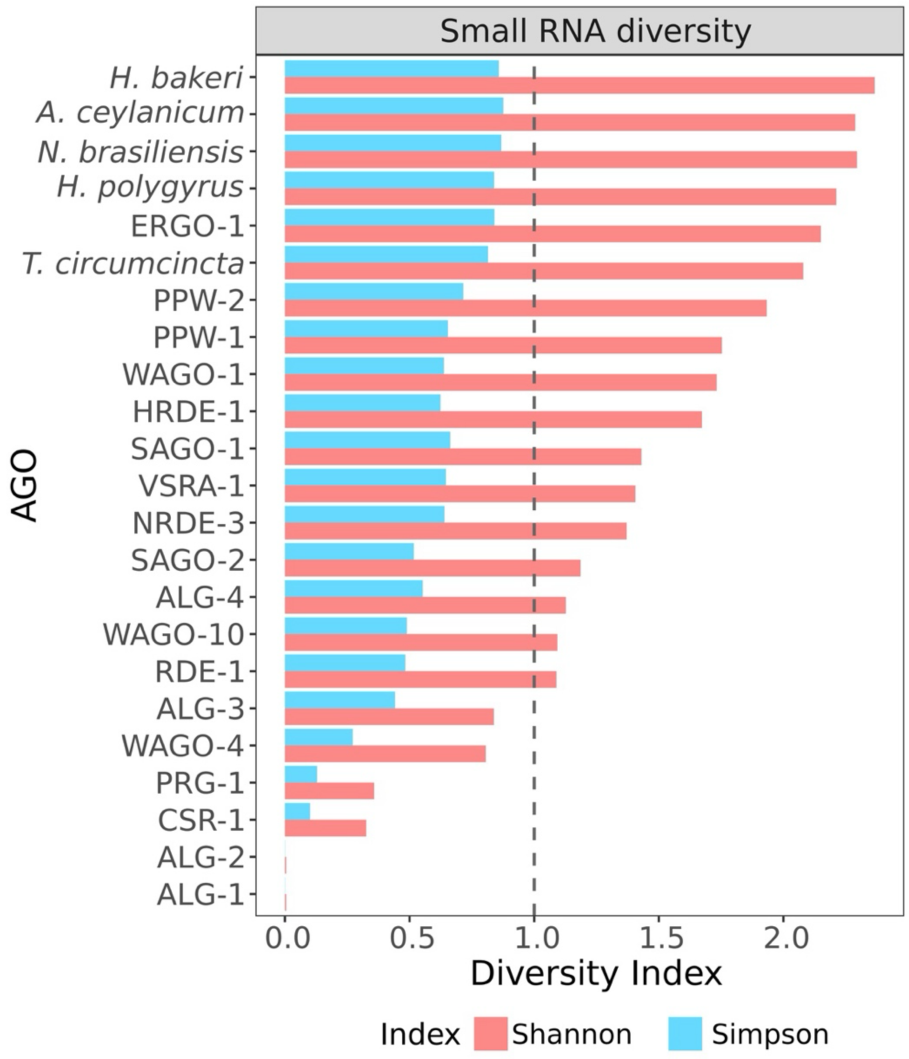

Figure S7. Small RNA diversity in *C. elegans* AGOs and Strongylida parasites. Small RNA diversity represented by Shannon and Simpson indices in *C. elegans* AGOs and exWAGO in Strongylida parasites. The grey dotted line indicates the maximum value (1) of the Simpson index.

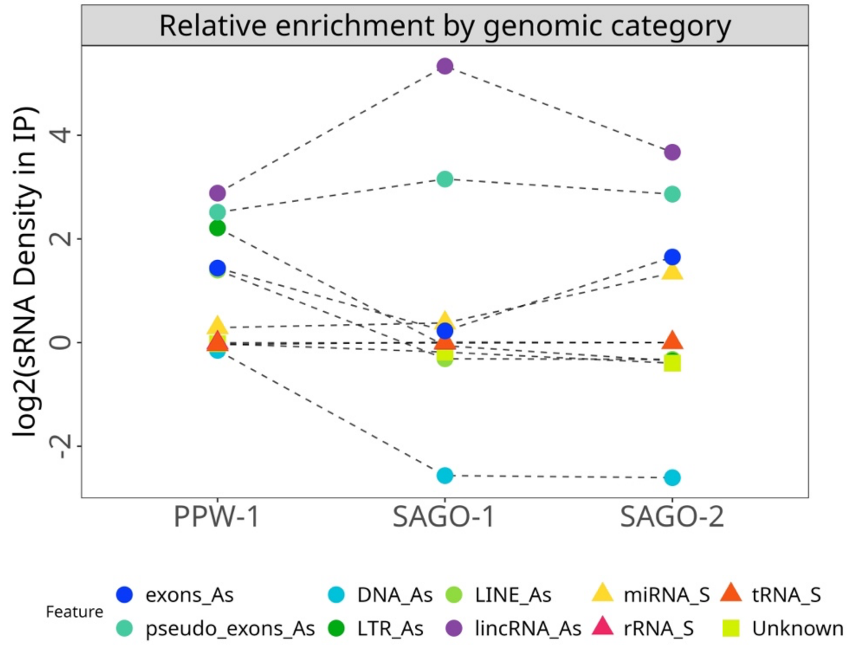

Figure S8. Relative enrichment by genomic categories in exWAGO orthologs in *C. elegans*. sRNA density was estimated using the percentage of Counts per Million (CPM) of enriched regions after differential expression analysis against input, divided by the genomic percentage per genomic category (log2-transformed; a pseudocount of 1 was added to both the numerator and denominator). The three exWAGO orthologs in *C. elegans* show consistent enrichment for antisense-derived lincRNAs and pseudogenes (considering only their respective exons).

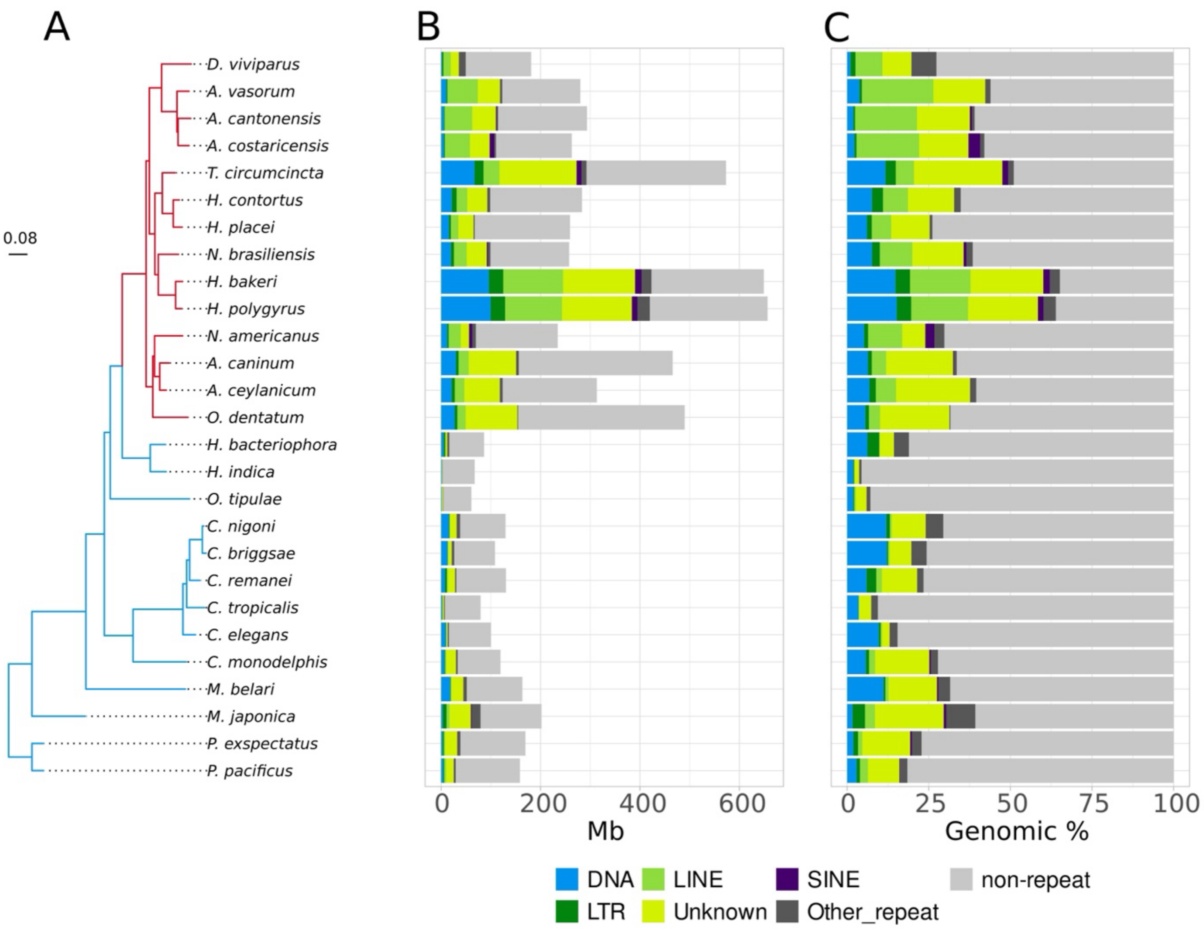

Figure S9. Repeat landscape in clade V nematodes. Repeat annotation after removing overlaps between elements of the same class. Maximum likelihood phylogeny inferred with a concatenated alignment of 443 complete BUSCO proteins present in the 27 nematode genomes. Red and Blue branches represent nematode species within the Strongylida and Rhabditida orders, respectively. The tree scale bar corresponds to 0.08 amino acid substitutions per site (A). Repeat span by class is shown in Mb. The category classified as non-repeat (see legend) represents all the genomic bases that were not annotated as TE or repeat. The Other repeat category represents the genomic bases annotated as Simple repeat or Low complexity repeat. RC/Helitron and DNA/MITE were collapsed within the DNA class (B). Repeat span by class as a genomic proportion (C).

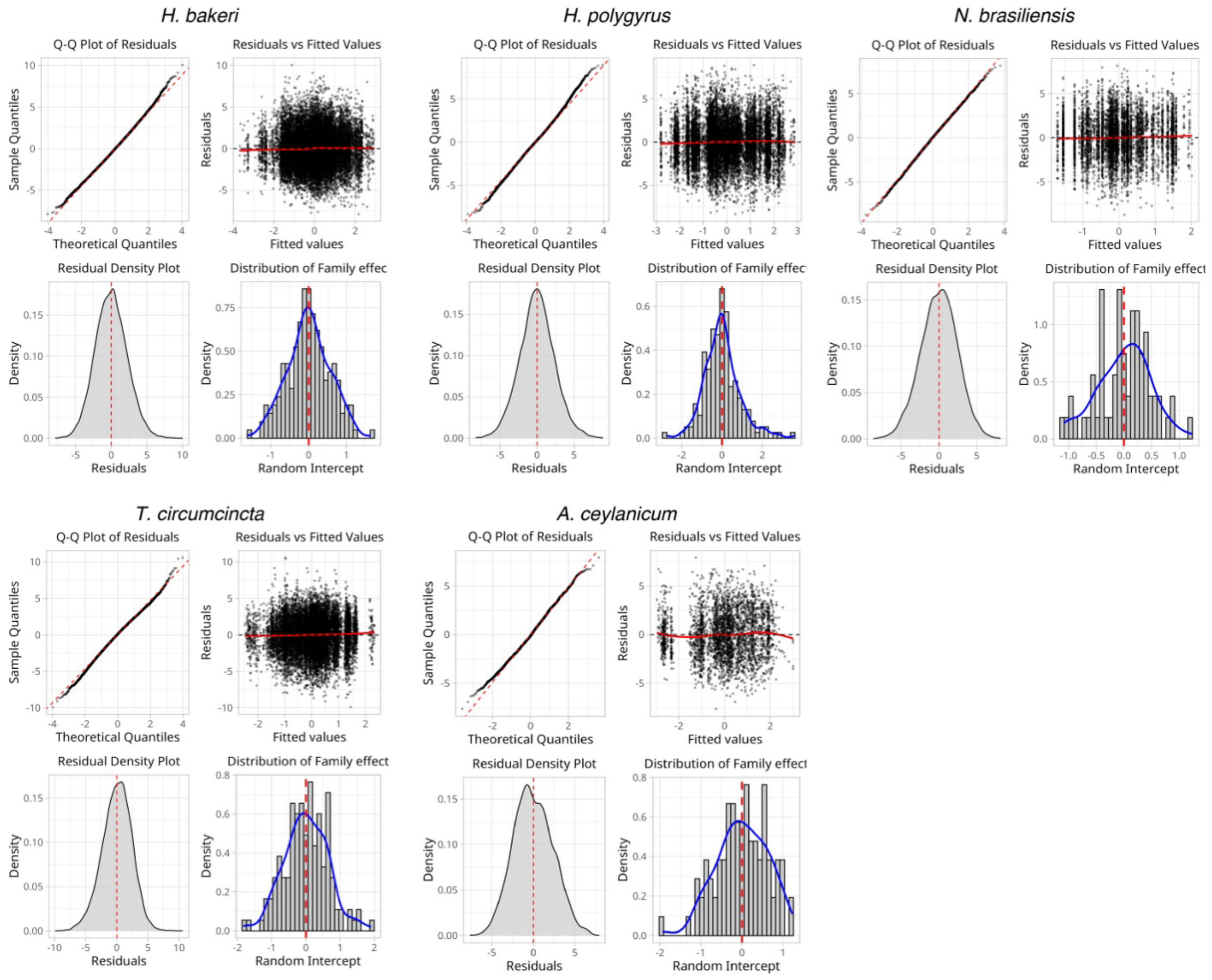

Fig S10. Generalised linear mixed model diagnostics for LTR-derived exWAGO guide production in Strongylida. Each panel shows diagnostic plots evaluating the fit of GLMMs, for LTR retrotransposons and modelled log2-centred expression of antisense sRNAs as the response variable. (1) Q–Q plot of residuals with theoretical normal distribution; (2) residuals vs. fitted values; (3) residual density plot; and (4) distribution of random intercepts for transposon family.

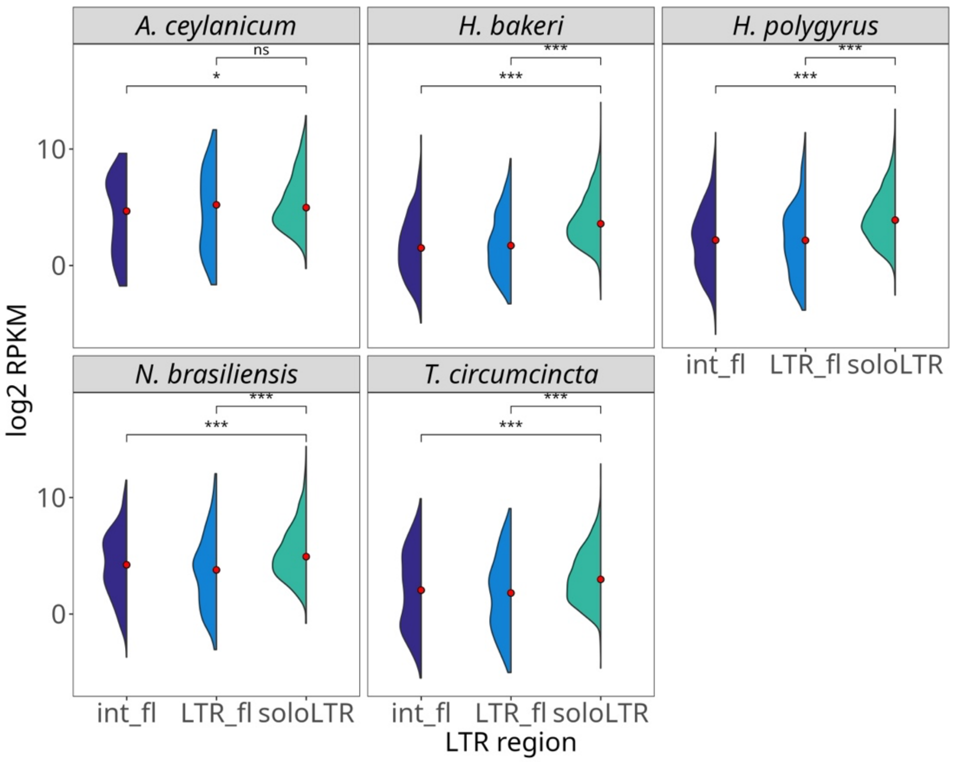

Fig. S11. Comparison of exWAGO guide production between full-length LTRs, considering their internal (int_fl) and LTR (LTR_fl) regions against soloLTRs in Strongylida parasites. The x-axis shows the distribution per category, and the y-axis shows log2 reads per kilobase per million (RPKM), for LTRs enriched in exWAGO guide production. The red dots inside each distribution represent the median value per category. A one-tailed Wilcoxon Rank Sum test was used for comparisons (ns not-significant; * p-value < 0.05).

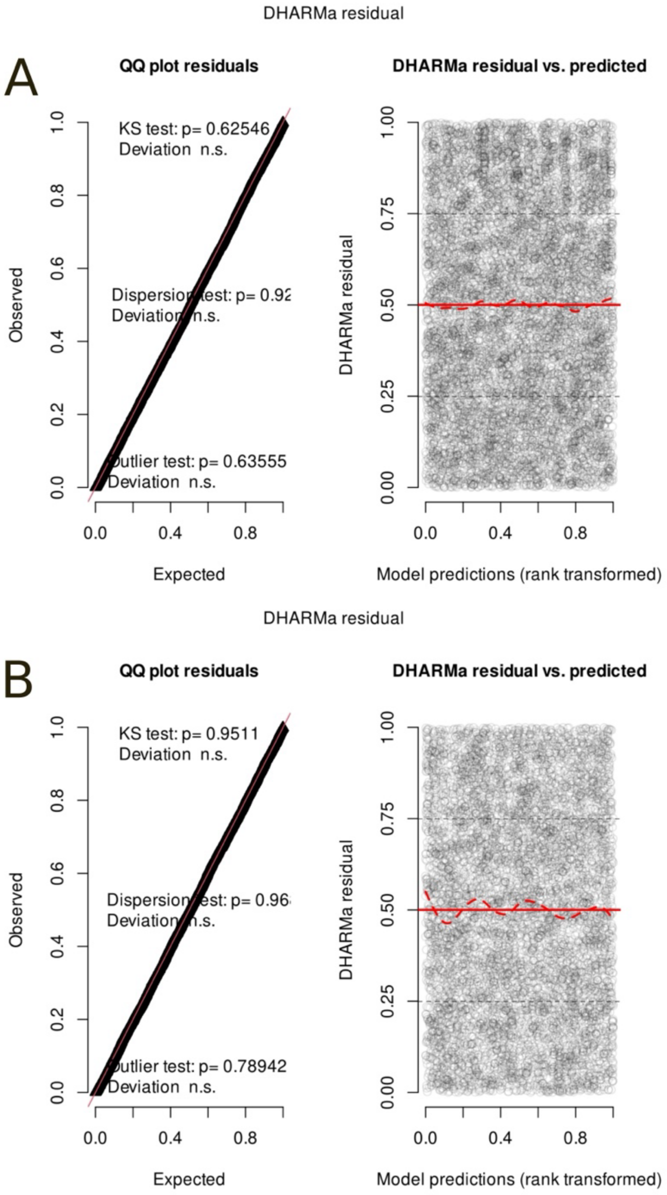

Fig. S12. Diagnostic residual plots for the binomial generalised linear model assessing secretion of exWAGO-bound LTR-derived sRNAs. Q–Q plots of simulated scaled residuals against the expected uniform distribution, and residual vs. predicted values are shown for vesicular exWAGO (A) and non-vesicular exWAGO (B). Kolmogorov–Smirnov goodness-of-fit test shows no significant deviations from the theoretical distribution ($p > 0.05$ in both exWAGO forms). Dispersion and outlier tests indicate no overdispersion or outlier inflation ($p > 0.05$). The red line indicates the 1:1 line. Residuals vs. predicted values, showing no systematic pattern or evidence of misfit. The dashed red line represents a smoothed LOESS fit of the residuals, while the solid red line shows the expected mean residual value.

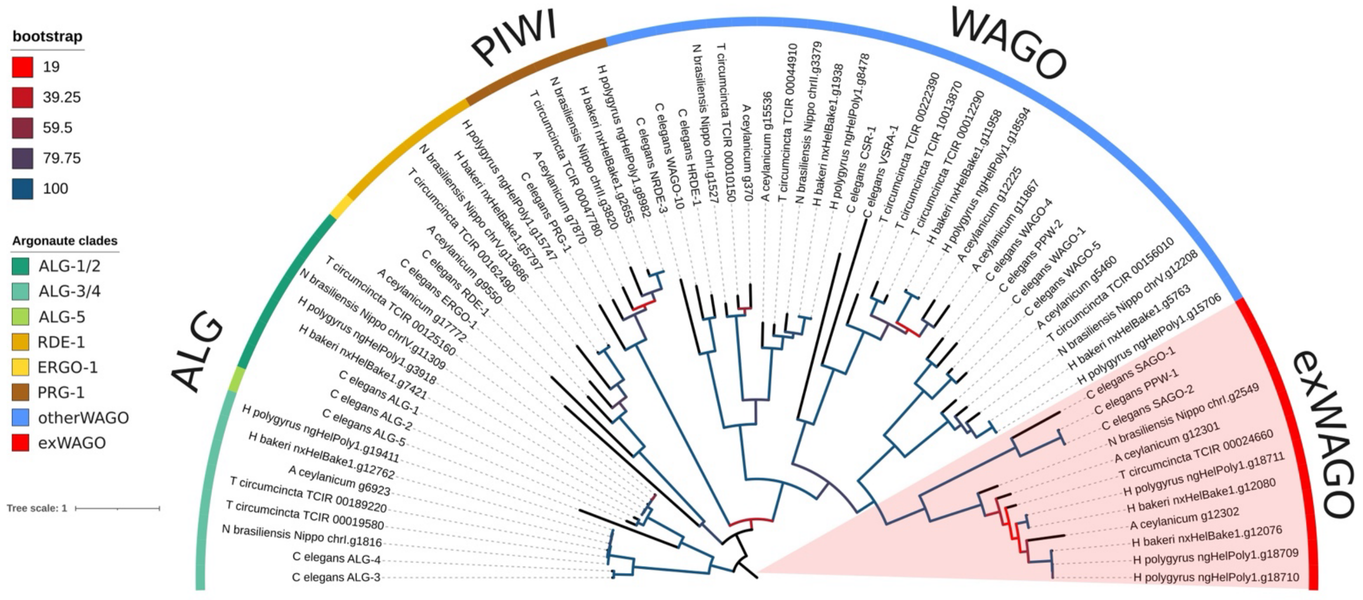

Figure S13. Phylogenetic relationship of *C. elegans* and selected Strongylida Argonautes. Maximum-likelihood phylogeny of Argonaute (AGO) proteins from *C. elegans* and selected Strongylida parasites. Branch colours indicate bootstrap support (see scale). AGOs are classified into canonical Argonaute clades based on phylogenetic position: ALG (ALG-1/2, ALG-3/4, ALG-5), PIWI (PRG-1), and WAGO. The WAGO clade is subdivided into other WAGOs (blue) and the exWAGO clade (red). The exWAGO clade comprises *C. elegans* SAGO-1, SAGO-2, and PPW-1 together with orthologous sequences from parasitic species.
